# Insight into Chemical Recycling of Flexible Polyurethane Foams by Acidolysis

**DOI:** 10.1021/acssuschemeng.1c07911

**Published:** 2022-01-11

**Authors:** Maja Grdadolnik, Ana Drinčić, Ana Oreški, Ozgun Can Onder, Petra Utroša, David Pahovnik, Ema Žagar

**Affiliations:** Department of Polymer Chemistry and Technology, National Institute of Chemistry, Hajdrihova 19, Ljubljana SI-1000, Slovenia

**Keywords:** chemical recycling, flexible polyurethane foam, microwave chemistry, sustainable chemistry, waste prevention

## Abstract

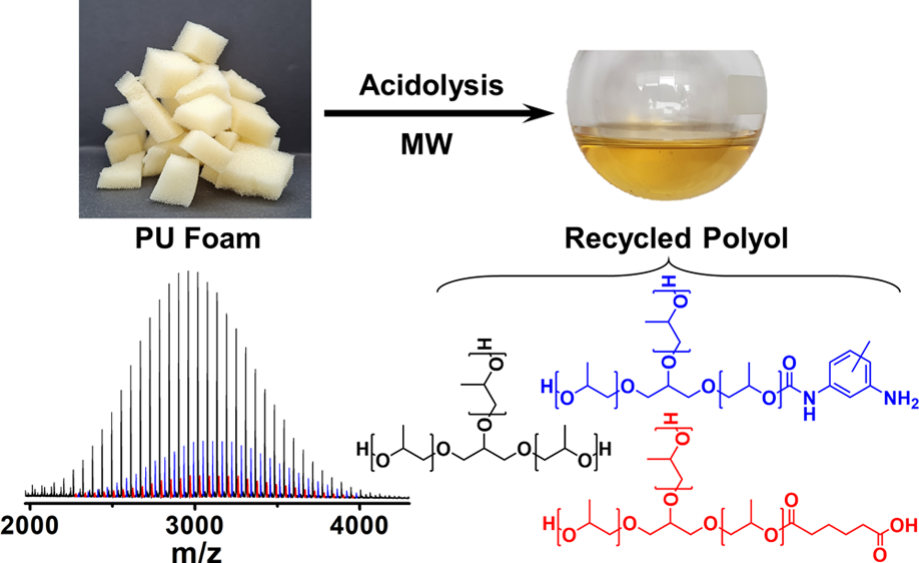

Acidolysis is emerging as a promising method for recycling polyurethane foam (PUF) waste. Here, we present highly efficient acidolysis of PUFs with adipic acid (AA) by heating the reaction mixtures with microwaves. The influence of experimental conditions, such as reaction temperature, time, and amount of the degradation reagent, on the polyol functionality, molecular weight characteristics, the presence of side products, and the degree of degradation of the remaining PUF hard segments was studied by matrix-assisted laser desorption/ionization time-of-flight mass spectroscopy (MALDI-TOF MS), nuclear magnetic resonance (NMR), size-exclusion chromatography (SEC) coupled to a multidetection system, and Fourier transform infrared (FT-IR) spectroscopy. The purified recycled polyols were used for the synthesis of flexible PUFs. The morphology and mechanical properties of the PUFs show that the degree of functionalization of the polyol by the carboxylic end groups, which is higher for larger amounts of AA used to degrade the PUFs, significantly affects the quality and performance of the flexible PUFs from the recycled polyols.

## Abstract 2

Reaction conditions of acidolysis of polyurethane foams determine the recycled polyol end-group functionality, which affects the polyurethane foam synthesis and properties.

## Introduction

Polyurethane foams (PUFs) are used in a variety of comfort applications or as thermal and acoustic insulation materials. Global production of PUFs is estimated to increase to 12.7 million tons by 2024.^1^ For this reason, recycling of PUFs is important from both economic and environmental points of view.^2,3^ Because PUFs are thermoset polymers with a cross-linked structure, mechanical recycling of PUF waste is not the most appropriate solution.^4^ For this reason, the chemical recycling of PUFs has recently become an interesting research topic.^5−11^ Chemical recycling of polyether polyol-based PUFs is based on the cleavage of the urethane bonds, leaving ether groups in polyether polyol intact. Several recycling technologies have been developed with different mechanisms of urethane bond degradation. Glycolysis^12−25^ and acidolysis^9,26−29^ have been successfully used at the industrial scale, hydrolysis^30,31^ has been introduced at the pilot scale, while aminolysis^32−35^ and phosphorolysis^36−38^ have been developed only at the laboratory scale. Chemical recycling of PUFs is carried out at high temperatures, mainly by conventional heating, although there are some reports on microwave (MW)-assisted degradation processes, especially in the case of glycolysis, where MW heating shortens the reaction time and improves the reaction yield.^17,19^ Theoretically, all recycling technologies for polyether-based PUFs lead to hydroxyl-functionalized polyether polyol and oligourea hard segments end-capped with the applied degradation reagent.^14,17,19^ Unfortunately, the existing methods of chemical recycling of PUFs mainly suffer from incomplete and/or nonselective degradation of urethane linkages, as partial cleavage of urea groups in the hard segments also occurs. For this reason, none of the PUF recycling methods is able to produce high-quality virgin-like polyols that would allow foam manufacturers to produce new flexible PUFs exclusively from the recycled polyol (RP) without compromising their properties.^22,23,39,40^

From an industrial point of view, acidolysis is emerging as a promising method for recycling PUF waste as it can be carried out without a medium or in a virgin polyol (VP) medium, in which the residues of the PUF hard segments are mostly insoluble, facilitating the isolation of polyol. Acidolysis of the urethane group was first performed on low-molecular-weight compounds using various carboxylic acids.^41^ It was found that the degree of acidolysis of ethyl carbamate and its derivatives depends on the type of the *N*-substituent and the type of carboxylic acid used. Acidolysis was also performed on a linear model polyurethane synthesized from methylene diphenyl diisocyanate and 1,4-butanediol using an excess amount of adipic acid (AA) at elevated temperatures in nitrobenzene solution, whereby its molecular weight decreased from 29.3 to 4.9 kDa after 6 h.^5^ H&S Anlagentechnik from Germany and Dendro from Poland have introduced chemical recycling of flexible PUFs on an industrial scale by using an acidolysis process based on conventional heating at 230 °C for 12 h.^9^ They argue several environmental and economic advantages, as the production cost of RP is 25–30% lower than the market price of the original polyether polyol. The obtained product mixture is characterized by the determination of hydroxyl, amine, and acid numbers and viscosity, but its exact chemical, structural, and molecular weight characteristics are not available, which makes it difficult to assess the extent of acidolysis and the quality of the recycled product. Recently, acidolysis of flexible PUF waste (a mixture of different PUF types) with diacids (PUF/diacid weight ratio of 4.5–5.5) in bulk under an inert atmosphere by conventional heating was reported.^26−28^ After 5 h, the resulting viscous polyol was discharged from the reactor and reused without any purification as a partial substitute (up to 30 wt %) of the primary polyol for the production of new rigid and flexible foams^26,27^ as well as polyurethane adhesives and coatings for wood.^28,29^ It is reported that the stiffness of the flexible PUFs increases with an increasing RP content, which was attributed to the presence of aromatic components in the RP. In addition, the authors investigated the effects of reaction conditions (reaction temperature and time, and the weight ratio between PUF and the degradation reagent) on the properties (hydroxyl number and acid value) of RPs and hypothesized that both thermal degradation and acidolysis are the mechanisms involved in the degradation of PUFs.^26−29^

In this work, we present a highly efficient MW-assisted acidolysis process for the conversion of PUFs into polyether polyol in less than 1 h reaction time. Our main objective was to evaluate acidolysis as one of the emerging chemical recycling processes in terms of RP quality, type of PUF hard segment residues, and ease of RP isolation, depending on the experimental conditions used. The RPs were purified to remove the low-molecular-weight byproducts and used for the synthesis of new flexible PUFs, where we investigated the influence of the end-group functionality of the purified RPs on the PUF polymerization process and ultimately on the structural and mechanical properties of the synthesized flexible PUFs.

## Experimental Section

### Degradation of PU Foams by Acidolysis

PUF was cryogenically ground using a vibratory ball mill (Tehtnica Millmix 20 Domel, Slovenia). In a typical depolymerization procedure, a mixture of ground PUF, AA, and VP ALCUPOL® F-5611 or ALCUPOL® F-4811 was put into a 30 mL glass vessel together with a magnetic stirrer. The vessel was sealed with polytetrafluoroethylene-coated silicone septa. Acidolysis of PUF was performed in a VP medium at a weight ratio of PUF to medium of 6/3 or without the medium (4 g of PUF). In the case of the 6/3 PUF/medium, a preheating step was necessary to ensure partial liquefaction of the PUF and sufficient stirring of the reaction mixture during the main heating step. The preheating step includes heating of the reaction mixture in 3 min to 175 °C, homogenizing it, and then subjecting it to the main heating step, which consists of heating the reaction mixture to a predetermined temperature (210, 220, and 230 °C) in a period of 5 min and maintaining this temperature for a defined time (15, 30, and 40 min). In case of acidolysis in bulk, good mixing was ensured by additional preheating steps, that is, heating of the reaction mixture in 3 min to 175 °C for 10 min and then to 190 °C for 3 min. During each step, the reaction mixture was homogenized manually. Finally, the reaction mixture was subjected to the main heating step for 30 min at 230 °C. AA was added in 1.1, 2.0, or 3.0 molar equivalents per PUF urethane group. Prior to MW-assisted degradation, the reaction mixture was purged with nitrogen to prevent oxidation of the amine-functionalized product, which would lead to the darkening of the reaction mixtures.^42,43^ The vials were then placed in a laboratory MW reactor Monowave 400 (Anton Paar GmbH, Austria) equipped with temperature and pressure sensors and a video camera. After completion of the degradation experiment, the reaction vessel was rapidly cooled by a stream of compressed air. The resulting reaction mixtures were centrifuged at 9000 rpm for 10 min. The upper polyol phase was isolated (purified) and further analyzed by ^1^H nuclear magnetic resonance (NMR), Fourier transform infrared (FT-IR) spectroscopy, size-exclusion chromatography coupled with ultraviolet, multi-angle light scattering, and refractive index (SEC/UV-MALS-RI) detectors, and matrix-assisted laser desorption/ionization time-of-flight mass spectroscopy (MALDI-TOF MS). The polyol remaining in the lower solid phase consisting mainly of the residues of the PUF hard segments was extracted with EtOAc. Thus, isolated RP was further purified by liquid–liquid extraction to remove the low-molecular-weight side products from the polyol. For this purpose, the solution of the polyol in ethyl acetate (EtOAc, 1 g mL^–1^) was washed with 0.1 M HCl, followed by pure water using a separatory funnel. Finally, EtOAc was removed from the RPs by evaporation on a rotary evaporator at 60 °C to obtain purified RPs.

### Synthesis of Flexible PUFs

Flexible PUFs were synthesized by the standard procedure using variable amounts of purified RPs, where 50 or 100% of VP was replaced by RP. A mixture of VP and RP was placed in a 50 mL cup, to which catalysts, surfactant, and demineralized distilled water as a blowing agent were added. The mixture was homogenized with a mechanical stirrer (Eurostar 40 digital, IKA, Germany) for 5 min at 2000 rpm. Then, an appropriate amount of toluene diisocianate (TDI80/20, TDI index of 107) was added to the mixture and homogenized again for 5–10 s. The mixture was quickly poured into a custom-made cardboard mold (8.5 × 8.5 × 8.5 cm; 6.14 dL). The foams were stored in a dark and dry place for 72 h to cure. Afterward, test specimens (2.5 × 2.5 × 1 cm) were cut for the analysis of mechanical properties using a dynamic mechanical analyzer (DMA).

## Results and Discussion

### PUF Acidolysis at Different Temperatures and Time

**Scheme 1:**
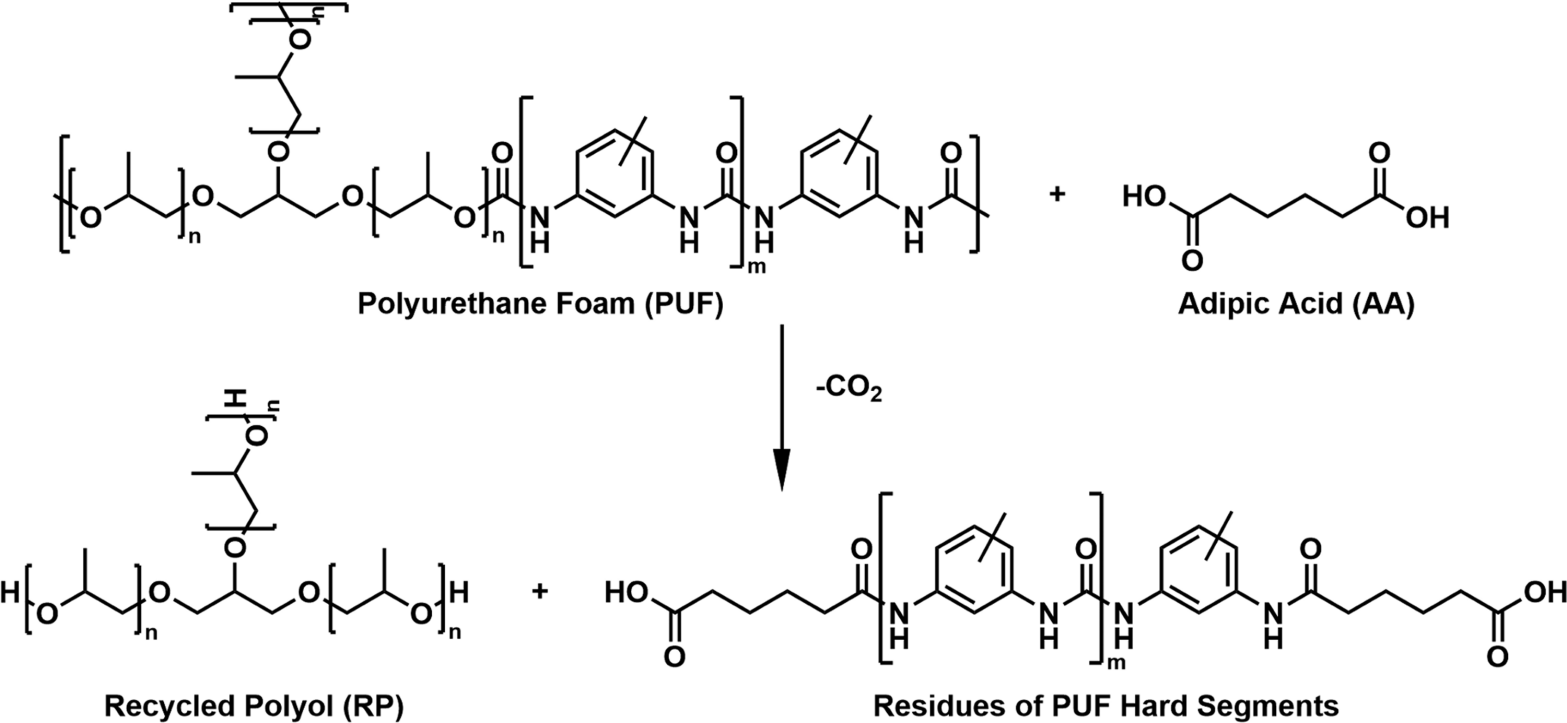
Reaction Scheme of Acidolysis of PUF with AA Leading to RP and Residues of PUF Hard Segments Terminated with AA

Acidolysis with AA was initially performed on PUF consisting of poly(propylene oxide)-based (PPO) homopolyether three-arm-star polyol with a glycerol core to facilitate characterization of the products obtained and the type of the polyol end groups by MALDI-TOF mass spectrometry. The reaction mixtures were heated by MW, and VP was used as a medium to facilitate stirring with a magnetic stirring bar and to prevent local overheating of the PUF. Compared with acidolysis performed by conventional heating, MW-assisted acidolysis could be carried out in a much shorter reaction time, that is, ∼30–40 min instead of several hours.^9,26−29^ Acidolysis of PUF with AA at a molar ratio of AA to PUF urethane groups of 1.1 (PUF/AA weight ratio of 9.4/1.0) and a weight ratio of PUF to VP medium of 6/3 was performed at different reaction temperatures (210, 220, and 230 °C) and reaction times (15, 30, and 40 min) (Table S1; entries 1–9). The molar ratio between AA and the PUF urethane groups of 1.1 (−COOH/–NHCOO– = 2.2) theoretically ensures a complete release of polyether polyol from the PUF with concomitant formation of carbon dioxide and oligourea hard segments terminated with carboxyl groups of AA moieties attached to the hard segments via amide bonds (Scheme 1).

Even after only 15 min of reaction time, the resulting product mixtures were completely soluble in dimethylsulfoxide (DMSO) regardless of the reaction conditions used, indicating a high degree of decomposition of the PUF network. The successful PUF acidolysis was confirmed by ^1^H NMR by the appearance of the amide (−NHCO−) signals between δ 8.95 and 9.88 ppm corresponding to the partially and fully amidated toluenediamine (TDA) and the terminal amidated aromatic isomers of the oligourea segments (Figures S1 and S2). The obtained product mixtures were then centrifuged to separate the viscous upper polyol phase from the lower solid phase consisting mainly of the residues of the PUF hard segments (Figure S3A,D). The resulting RPs were analyzed for their molecular weight characteristics, the content and type of nonhydroxyl end groups, and the content of TDA, the presence of which is undesirable in polyol because of its toxicity.

The SEC/UV-MALS-RI chromatograms of the obtained RPs show the presence of polyol at 9.4 min and the absence of any high-molecular-weight species, confirming a high degree of PUF network decomposition (Figure S4). Low-molecular-weight residues of the soluble PUF hard segments in the RPs (mainly TDA isomers and in trace amount monoamidated TDA derivatives and urea) are detected at longer elution times (22–37 min), mainly by the UV detector because of the low concentrations of these side products in the RPs (Figure S4).

**Figure 1:**
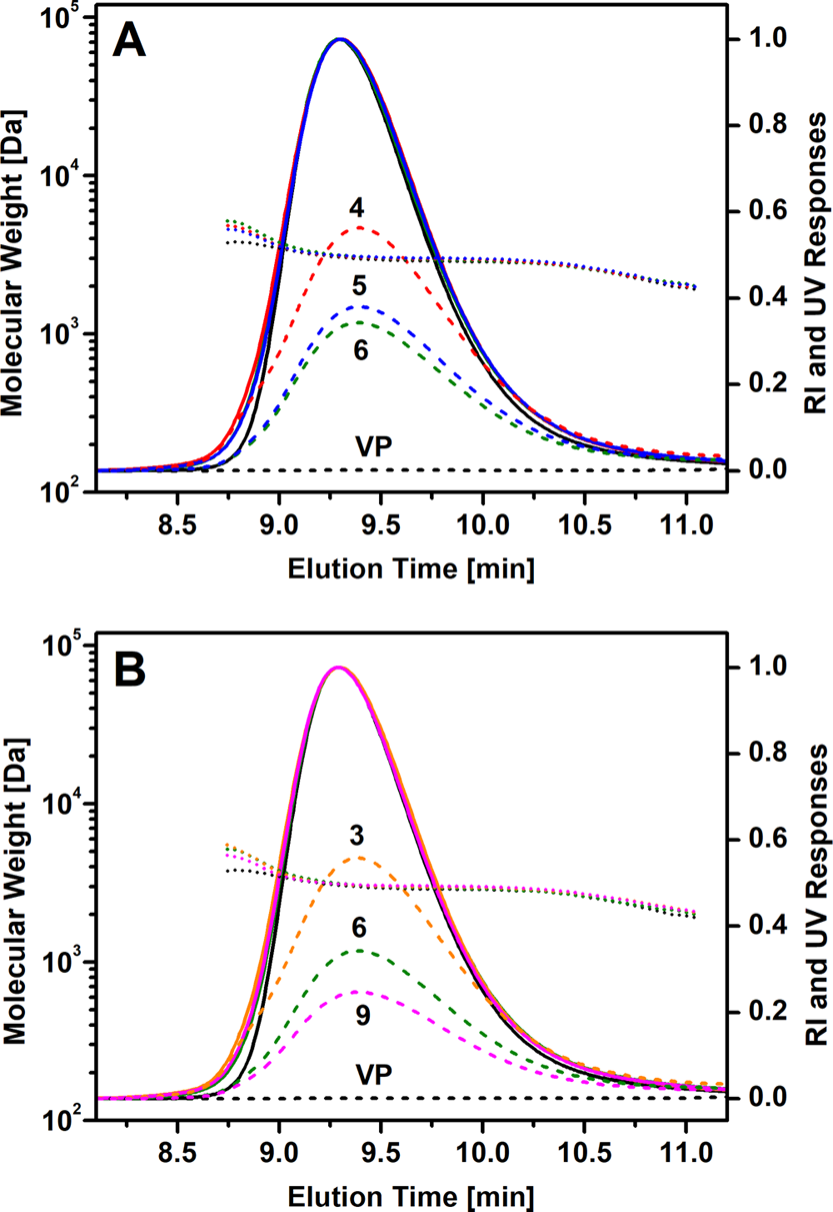
SEC/UV-MALS-RI chromatograms of RPs obtained by PUF acidolysis at different reaction conditions: (A) 220 °C: 15 min (Entry 4), 30 min (Entry 5), 40 min (Entry 6) and (B) 40 min: 210 °C (Entry 3), 220 °C (Entry 6), 230 °C (Entry 9) together with the SEC/UV-MALS-RI chromatogram of VP of the same type. The solid and dashed lines represent the RI and UV (λ = 280 nm) detector responses, respectively, while the dotted lines represent molecular weight as a function of elution time.

With increasing reaction temperature and time, the SEC/UV-MALS-RI chromatograms of RPs show a continuous decrease in the ratio of UV to RI responses of the polyol, which can be attributed to an increasingly higher degree of degradation of urethane groups and consequently a decreasing fraction of UV-active polyol chains functionalized with aromatic end groups (Figure 1). Regardless of the acidolysis temperature and time, the molecular weight characteristics (weight and number average molecular weights and dispersity) of the RPs are comparable to those of the VP, from which the foam was prepared because they differ only in the type of the RP end groups (Table 1; entries 1–9).

**Table 1:** Molecular Weight Characteristics (Weight Average Molecular Weight; *M*_w_, and Dispersity; *Đ* = *M*_w_/*M*_n_) and the Content of Nonhydroxyl End Groups (Carboxyl and Aromatic Amine) and TDA in the RPs Obtained by Acidolysis of PUFs at a Weight Ratio of PUF/VP of 6/3 or without the VP Medium (in Bulk) at Different Temperatures, Times, and Molar Ratios of AA to the Urethane Group

entry	PUF type	AA/urethane group (mol/mol)	*T* (°C)	*t* (min)	nonhydroxyl end-group content^c^ (mol%)	–COOH content^d^ (mol%)	–NH_2_ content^e^ (mol%)	TDA content^f^ (wt %)	*M*_w_ (Da)	*Đ*
	ALCUPOL® F-5611								3000	1.02
	ALCUPOL® F-4811								3500	1.02
1	PUF5611	1.1	210	15	15.7			2.1	3200	1.05
2	PUF5611	1.1	210	30	12.3			2.9	3200	1.03
3	PUF5611	1.1	210	40	11.3	5.7	5.8	3.1	3200	1.03
4	PUF5611	1.1	220	15	12.3			2.2	3100	1.03
5	PUF5611	1.1	220	30	8.7	4.1	4.3	3.2	3100	1.03
6	PUF5611	1.1	220	40	7.3	4.3	3.2	3.6	3100	1.02
7	PUF5611	1.1	230	15	12.0			2.2	3100	1.03
8	PUF5611	1.1	230	30	8.3			3.6	3100	1.02
9	PUF5611	1.1	230	40	6.3	4.2	1.8	4.2	3100	1.02
10	PUF5611	2.0	220	30	12.3	8.1	2.4	2.4	3200	1.02
11	PUF5611	3.0	220	30	15.3	13.8	1.7	1.1	3300	1.02
12^a^	PUF5611-bulk	1.1	230	30	8.3	4.7	2.8	3.9	3200	1.03
13^b^	PUF4811	1.1	220	30	7.3	4.0	3.7	4.0	3600	1.02
14^b^	Post-Consumer PUF	1.1	220	30	7.3	2.7	4.1	4.1	3600	1.02
15	Recycled PUF5611	1.1	230	40	7.0	4.1	2.1	4.3	3100	1.02

a Acidolysis in bulk was performed with two preheating steps.

b In calculation of the contents of nonhydroxyl end groups (carboxyl and amine) and TDA, the molecular weight of 3.5 kg mol^–1^ and the chemical composition of the copolymeric polyol Alcupol F-4811 were taken into account.

c Calculated according to eq S1.

d Calculated according to eq S2.

e Calculated according to eq S3.

f Calculated according to eq S4.

**Figure 2:**
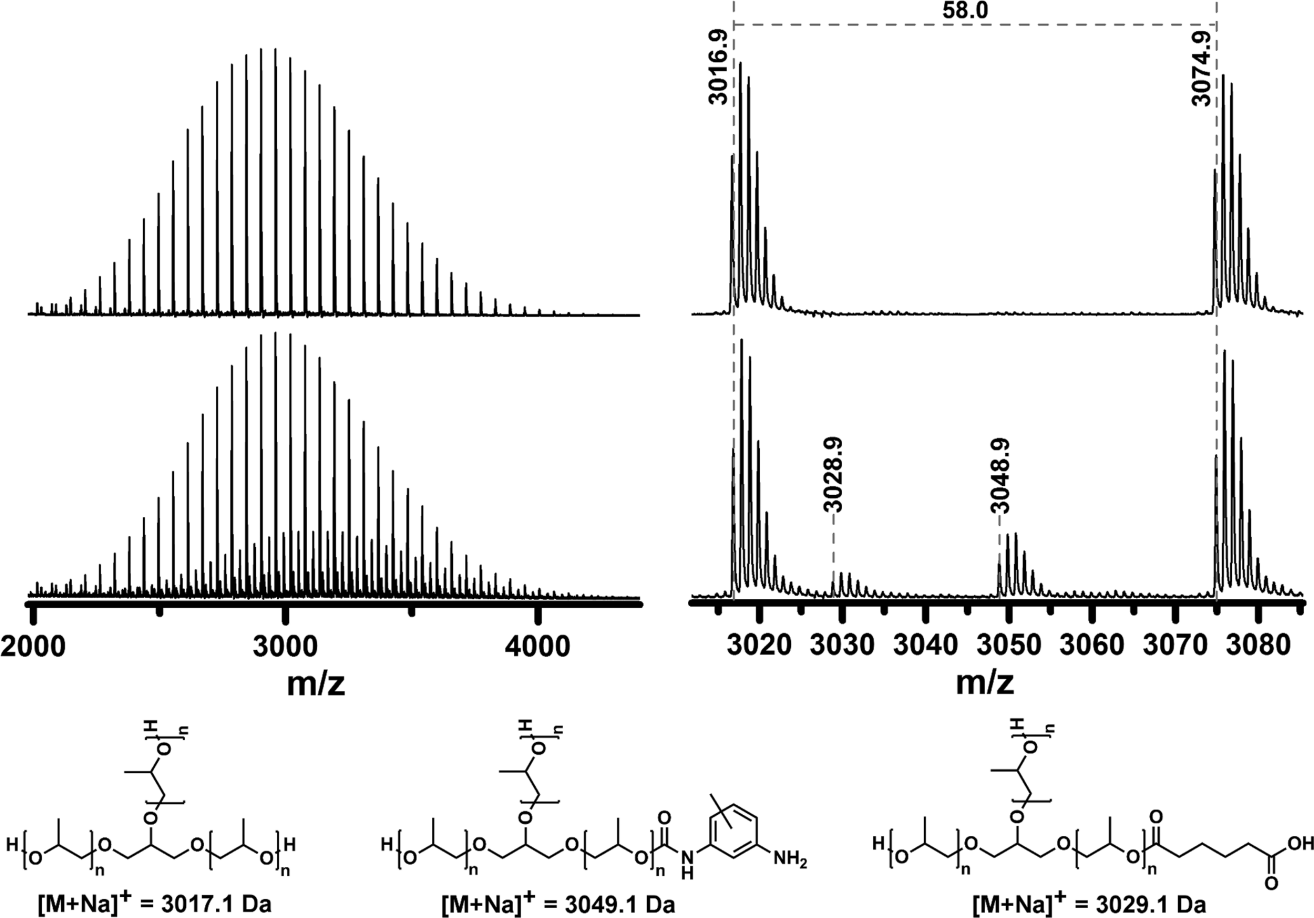
MALDI-TOF mass spectra of a typical RP isolated from the crude reaction mixture by centrifugation (bottom) and VP of the same type (top). The measured monoisotopic signals are denoted in the magnified regions of the mass spectra and are in good agreement with the calculated exact masses (M) ionized with the sodium ion for the proposed structures.

The MALDI-TOF mass spectra of all the RPs show the main peak population corresponding to the desired hydroxyl-functionalized polyol, and two much weaker peak populations because of the polyol chains terminated at one chain end by the aromatic amine (TDA moiety, attached to the polyol via the urethane group), resulting from incomplete degradation of the urethane groups, and by a carboxyl functional group, resulting from the esterification of a hydroxyl group of the polyol by AA (Figure 2).

**Figure 3:**
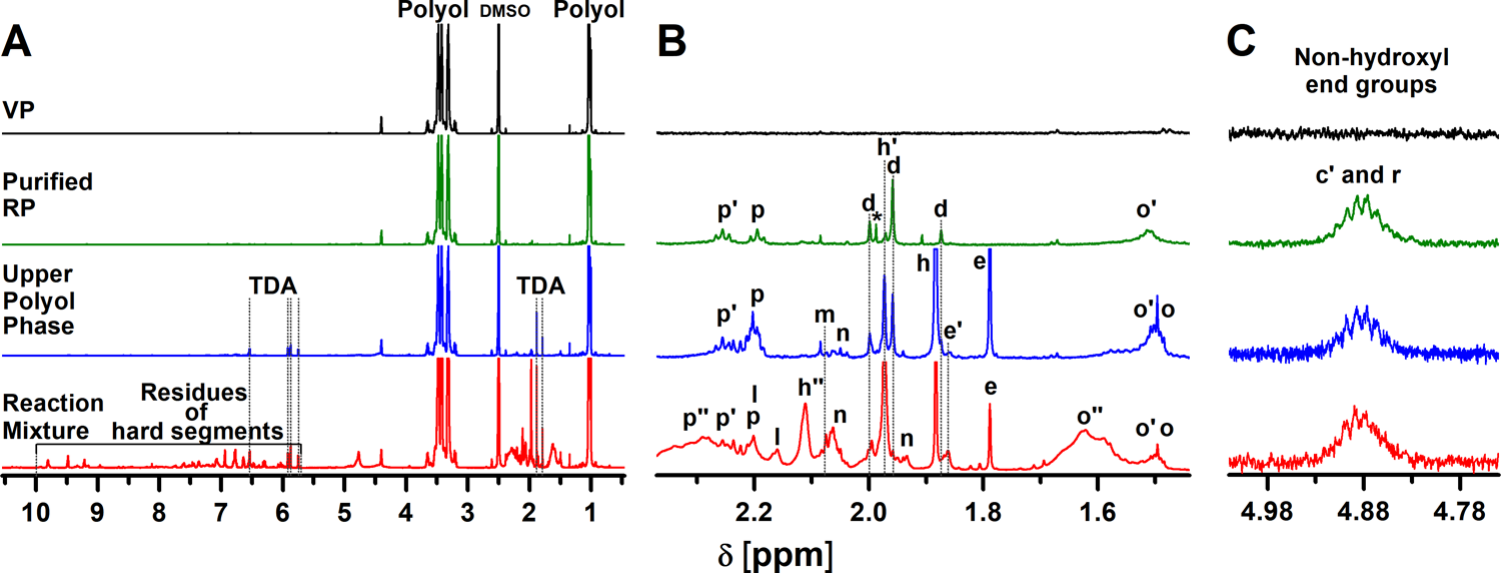
(A) Magnified ^1^H NMR spectra of the crude reaction mixture obtained after PUF acidolysis with AA at 220 °C for 30 min and a molar ratio of the AA/urethane group of 1.1, the isolated upper polyol phase after centrifugation of the crude reaction mixture, and the purified RP and VP of the same type, recorded in DMSO-*d*_6_, together with the (B) magnified region typical for the quantification of the nonhydroxyl end groups of the polyol. (C) Magnified region of the ^1^H NMR spectra (normalized to the polyol methyl group) of the same samples recorded in DMSO-*d*_6_ with added TFA, showing the overlapping signals of the polyol methyne group near the urethane and ester bonds. The peak assignment refers to the structures shown in Table S4.

The ^1^H NMR spectra of the isolated RPs show mainly the signals of the differently terminated polyol (d, p, p′, and o′), low-intensity signals of TDA isomers (e and h) soluble in the polyol and other impurities in trace amounts, such as urea (n) and partially amidated 2,6- and 2,4-TDA isomers (h′ and e′), which are finely dispersed in the polyol and do not settle during centrifugation (Figure 3A,B and Table S4). In the ^1^H NMR spectra recorded in DMSO-*d*_6_ with added trifluoroacetic acid (TFA), the representative signal attributed to polyol esterification by AA is found at δ 4.88 ppm for the polyol methyne signal (r) near the ester group, which overlaps with the polyol methyne signal (c′) near the urethane group (Figure 3C). For this reason, the exact degree of degradation of the urethane groups cannot be determined from this signal alone, but it is indicative of the content of the nonhydroxyl end groups of the RPs, which can be determined according to eq S1. The results show that at all temperatures, most of the urethane groups are degraded within the first 15 min of the reaction (Table 1; entries 1–9). As the reaction temperature increases or the reaction time is extended, the content of nonhydroxyl end groups decreases, but none of the reaction conditions used resulted in a completely hydroxyl-functionalized polyol.

Because in the ^1^H NMR spectra of the RPs isolated from the crude reaction mixtures, the aromatic methyl signals of the polyol amine end groups (three signals belonging to the residues of the TDA isomers marked with d) partially overlap with the monoamidated 2,4-TDA (h′) and 2,4-TDA (h), and moreover, the methylene signals of AA (p, p′, and o′) involved in the esterification reaction overlap with those involved in the amidation reaction (p″) and free AA (o, p), the exact content of aromatic amine and carboxyl end groups in the RPs cannot be determined (Figure 3B). Therefore, the polyols were purified by liquid–liquid extraction using EtOAc/0.1 M HCl (aq). In this way, TDA, partially amidated TDA, and urea were almost completely removed, as shown by SEC/UV-MALS-RI and ^1^H NMR (Figures S4, 3A,B). The content of carboxyl groups of RP was determined according to eq S2 from the proton signals denoted as p and p’ corresponding to the methylene groups of the AA residue adjacent to the carboxyl and ester groups, respectively, and the methyl signal (a) of the polyol (Figure 3B and Table S4). The content of amine functional groups of RP was determined according to eq S3 from the methyl signals of the aromatic amine end groups attached to the polyol via the urethane groups (denoted as d) and the methyl signal (a) of the polyol (Figure 3B and Table S4). The results show that with increasing temperature, the content of polyol chains terminated with amine end groups decreases (Table 1; entries 3, 6, and 9, Figure S5).

Acidolysis at a ratio of AA to urethane groups of 1.1 is not selective only for urethane bonds because the urea groups of the hard segments also participate in the reaction, leading to excessive formation of TDA. Indeed, with increasing temperature and time, the ^1^H NMR spectra of the lower solid phases (Figures S1 and S2) show an increasing intensity of the signals belonging to partially and fully amidated TDA derivatives at the expense of a decreasing intensity of the typical signals of oligourea hard segments. Consequently, the content of TDA in the isolated RPs, determined from the signal intensities of the methyl groups of the TDA isomers (e and h) and the methyl group of the polyol (a) according to eq S4, also increases in the same order (Table 1; entries 1–9). The RP phases thus contain between 2.1 and 4.2 wt % TDA, corresponding to conversion from 10.7 to 20.7 mol % of TDI used in PUF formulation to TDA. Because the content of nonhydroxyl end groups decreases only slightly with a concomitant increase in TDA formation, extending the reaction time beyond 40 min or increasing the reaction temperature above 220–230 °C can be considered ineffective in PUF acidolysis.

### PUF Acidolysis at Different Amounts of AA

When the amount of AA in the reaction mixture was increased to 2.0 and 3.0 equivalents per urethane group (i.e., PUF/AA weight ratios of 5.2/1.0 and 3.5/1.0, respectively, as typically reported in the literature),^9,26−29^ the extent of acidolysis of the hard segments also increases, similar to what was observed with increasing acidolysis temperature and time (Table 1; entries 5, 10, and 11). For example, at a PUF/AA weight ratio of 3.5/1.0, the hard segments were completely converted to di- and monoamidated TDA and free TDA after 30 min of PUF acidolysis at 220 °C, as shown by the ^1^H NMR spectrum of the obtained lower solid phase (Figure S6, bottom). In contrast to PUF acidolysis, which is carried out with 1.1 equivalents of AA per urethane group and leads to hard segment residues more or less dispersed in the polyol, acidolysis carried out with higher amounts of AA under otherwise the same reaction conditions results in better separation of the upper polyol phase from the PUF hard segment residues (Figure S3B and E vs A and D). Therefore, most of the polyol can be easily poured off from the crude reaction mixture, which greatly facilitate its isolation. Nevertheless, the yields of the reactions determined by isolating the RPs by dissolution in EtOAc and further purification are comparable (∼90%), regardless of the amount of AA used (Table S2).

**Figure 4:**
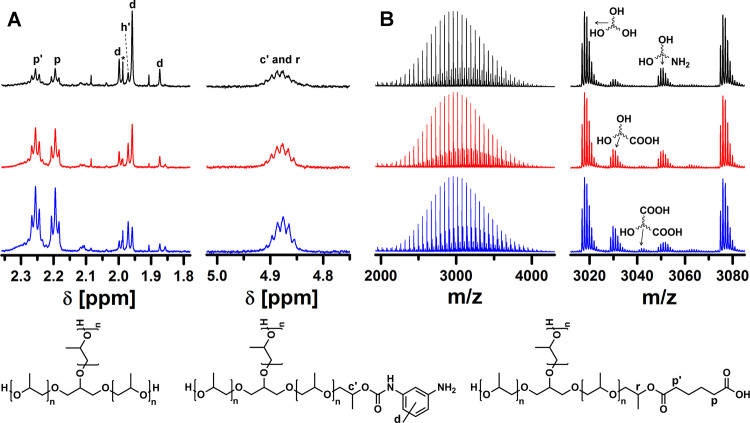
(A) ^1^H NMR spectra and (B) MALDI-TOF mass spectra of purified RPs recovered from PUF at 220 °C, 30 min with 1.1 (black), 2.0 (red), and 3.0 (blue) equivalents of AA per urethane group. ^1^H NMR spectra representing the magnified region between 4.75 and 5.03 ppm were recorded in DMSO-*d*_6_ with added TFA and are normalized to the polyol methyl group. Traces of EtOAc used as the extraction solvent and partially amidated 2,4-TDA in the RPs are indicated by asterisk and h′, respectively.

When acidolysis of PUF was performed at 220 °C for 30 min with higher amounts of AA, the degree of amidation of the released TDA improved, and consequently, the amount of free TDA in the polyol decreased from 3.2 wt % at 1.1 molar equivalents to 1.1 wt % at 3.0 molar equivalents of AA to the urethane group (Table 1; entries 5, 10, and 11, Figure S7; signals e and h). Interestingly, although the extent of degradation of the PUF hard segments increases with a higher amount of AA, the content of nonhydroxyl end groups in the polyol, as determined by ^1^H NMR, increases from 8.7 to 15.3 mol % for 1.1 and 3.0 AA equivalents per urethane bond, respectively (Table 1; entries 5, 10, and 11, Figure 4A; signals c′ and r). MALDI-TOF MS, ^1^H NMR, and FT-IR spectra of the polyol samples show that higher contents of polyol nonhydroxyl end groups are a consequence of the higher degree of esterification of the polyol hydroxyl end groups by AA (Figures 4 and S8). Increasing AA from 1.1 to 3.0 molar equivalents per urethane group results in a decrease in the content of aromatic amine end groups from 4.3 to 1.7 mol % because of the increased degree of urethane group degradation, while the content of carboxyl end groups increases from 4.1 to 13.8 mol % because of the higher degree of esterification of the polyol hydroxyl end groups so that some polyol macromolecules have even two chains terminated with carboxyl groups (Figure 4B). These results indicate that higher amounts of AA can reduce the content of TDA in the polyol and improve the degree of degradation of urethane groups, but at the expense of a higher degree of esterification of the polyol, so that the resulting RPs are terminated with carboxyl groups to a greater extent, although they have comparable molecular weight characteristics to VPs of the same type (Figures 4 and S8, Table 1).

### PUF Acidolysis in Bulk

Acidolysis of PUF at 1.1 equivalents of AA to urethane groups was also carried out in bulk without the use of a medium (Table 1; entry 12). In this case, difficulties were encountered in stirring the reaction mixture with a magnetic stirrer, resulting in local overheating of the foam. Therefore, acidolysis was performed in bulk by heating the reaction mixture in several steps, during which the reaction mixture was homogenized manually. PUF acidolysis performed in this way resulted in RP with comparable molecular weight characteristics and contents of TDA and nonhydroxyl end groups to those of RP obtained by PUF acidolysis in the VP medium (Table 1; entry 12 and Figures S9A, S10, S11A, and S12A), indicating the possibility of performing PUF acidolysis without the presence of a medium, but only if sufficient stirring of the reaction mixture is ensured.

### Acidolysis of Copolyether-Based PUFs

Next, the MW-assisted acidolysis method was used to degrade the PUF prepared from a commonly used copolyether three-arm-star polyol containing ethylene oxide and propylene oxide repeating units attached to a glycerol core. Compared to PUF5611, the acidolysis of copolyether-based PUF4811 in the corresponding VP medium leads to a better separation of the upper polyol phase from the residues of the PUF hard segments already at 1.1 equivalents of AA per urethane group (Figure S3C and F vs A and D). The results regarding the content of TDA and nonhydroxyl end groups in the recovered copolyether polyol are very similar to the values obtained for the recycled PPO-based RP (Table 1; entries 5 and 13 and Figures S9B, S11B). Moreover, the molecular weight characteristics of both RPs are comparable to those of the corresponding VPs, indicating successful recycling of PUFs from different types of (co)polyether polyols (Table 1; entries 5 and 13, Figure S12B). The optimal acidolysis procedure was also applied to recycle a postconsumer PUF4811 waste that contained additives such as dyes, calcium carbonate, and flame retardants. The ^1^H NMR spectrum of the RP obtained from the postconsumer PUF waste shows no signals attributable to additional impurities, while the TDA and nonhydroxyl end-group contents and molecular weight characteristics are comparable to those determined for the copolyether-based RP, from which the post-consumer PUFs were prepared (Table 1; entries 13 and 14, Figures S9B, S11B, and S12B).

### Synthesis of Flexible PUFs from RPs

The purified PPO-based RPs obtained from the PUF by acidolysis with 1.1 and 3.0 equivalents of AA per urethane group were further used to synthesize new flexible PUFs without adjusting the PUF formulation or synthesis conditions compared to the PUF prepared from 100% VP (Table S3). The hydroxyl numbers and acid values determined according to eqs S5, S6, and S7, water content, molecular weight, and structural properties of RPs are listed in Table S2. The two polyols used for PUF synthesis differ mainly in the content of carboxyl end groups in order to evaluate its influence on the polymerization process and the structural and mechanical properties of the synthesized flexible PUFs.

**Figure 5:**
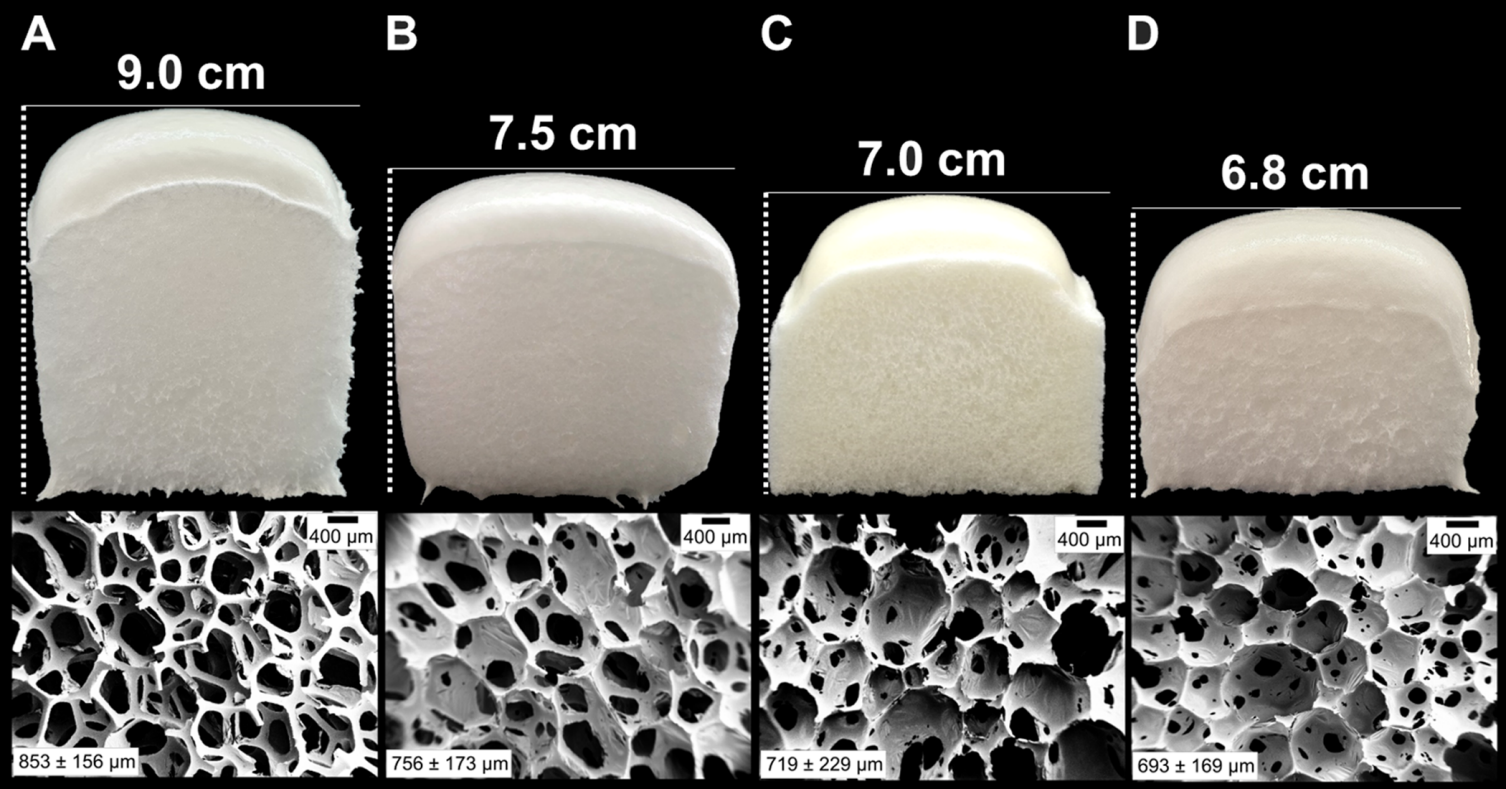
Photographs and cross-sectional images of PUFs prepared using the formulation shown in Table S3 with (A) 100% VP, (B) 50% RP containing 5.6 mol % carboxyl and 3.2 mol % aromatic amine end groups, (C) 100% RP containing 5.6 mol % carboxyl and 3.2 mol % aromatic amine end groups, and (D) 50% RP containing 14.0 mol % carboxyl and 1.6 mol % aromatic amine end groups. The average pore sizes in μm are given in the lower-left corners of the images.

The PUF prepared from VP has an open-cellular morphology (Figure 5A), while the PUFs prepared by replacing VP in the formulation with 50 and 100 wt % RP with 5.6 mol % carboxyl and 3.2 mol % aromatic amine end groups (obtained by PUF acidolysis with 1.1 mol equivalents of AA per urethane group) show a decreased average pore size and an increasingly closed-cell morphology (Figure 5B,C). The closed-cell morphology, as well as the decreased foam height and delay in cream and rise times, indicate a faster cross-linking reaction relative to the gas formation reaction, most likely due to the deactivation of the amine catalyst by the polyol carboxyl end groups through protonation. This effect was more pronounced when the RP content in the formulation was higher (100%) or when 50 wt % of VP was replaced by RP with a higher content (14.0 instead of 5.6 mol %) of carboxyl end groups obtained by PUF acidolysis with 3.0 molar equivalents AA per urethane group (Figure 5D).

The mechanical properties of the PUFs were investigated using compression tests (Table 2 and Figure S13). Compared to PUF synthesized from VP, PUFs synthesized from a progressively higher content of RP in the formulation show an increase in modulus and stress at 40% compression, which is attributed to a gradually more pronounced closed-cell morphology and higher PUF density. Because flexible PUFs are widely used cushioning materials, good recovery after prolonged compression is desirable. The compression set property, as a potential predictor of the height and load bearing loss sensitive to changes in the PUF network, was measured at 50% strain at 70 °C for 22 h and determined according to eq S8. The replacement of 50 wt % VP with RP containing 5.6 mol % carboxyl groups increased the compression set from 4.0 to 7.4%, indicating slightly lower durability of the recycled foam, but still within specifications for standard flexible foams.^44^ The PUF made from 100% RP of the same type shows modulus and stress at 40% compression comparable to the PUF made from 50% RP, but its quality in terms of nonrecoverable deformation has decreased significantly, as shown by the increase in the compression set from 7.4 to 16.7%. In the literature, for flexible PUF obtained by replacing 60 wt % VP with glycolysis-derived RP, a fivefold increase in the compression set is reported compared to PUF prepared entirely from VP.^25^ The increasing deformation of PUFs with increasing RP content in the formulations was previously attributed to the presence of low-molecular-weight impurities in the polyols, such as amine-terminated aromatic residues of hard segments and/or the glycol reagent in the case of glycolysis, which react with diisocianate and lead to stiffer PUFs enriched with hard segments.^25,39,40,45,46^

**Table 2:** Densities and Mechanical Properties of PUFs

polyol for PUF synthesis	PUF density (kg m^–3^)	compress. modulus (kPa)	stress at 40% compression (kPa)	compression set (%)
ALCUPOL® F-5611	27.1 ± 1.8	18.1 ± 4.7	1.85 ± 0.27	4.0 ± 1.5
AC1.1-RP50	28.4 ± 0.4	25.6 ± 1.1	2.68 ± 0.16	7.4 ± 1.5
AC1.1-RP100	29.7 ± 0.4	26.6 ± 5.2	2.79 ± 0.27	16.7 ± 2.6
AC3.0-RP50	30.8 ± 0.1	39.0 ± 1.0	2.78 ± 0.17	14.9 ± 0.6

In our case, the low-molecular-weight impurities were almost completely removed from the RPs, as shown by SEC/UV-MALS-RI and ^1^H NMR (Figures S4 and 3A). Therefore, the enhanced compressive performance of our PUFs made of purified RPs is more likely a consequence of the effect of the polyol carboxyl end groups on the relative rates of cross-linking and foaming reactions, which determine the morphology of the PUFs and ultimately their mechanical properties. Indeed, the PUF prepared with the same amount of RP (50%) but with a threefold higher content of carboxyl groups exhibits a significantly higher compressive modulus (39.0 vs 25.6 kPa) and a compression set (14.9 vs 7.4%). These results suggest that not only the low-molecular-weight functional impurities but also the type and content of nonhydroxyl end groups in RPs have a profound effect on the quality and performance of flexible PUFs synthesized from RPs.

Finally, PUFs synthesized from 100% RP were again subjected to acidolysis under optimal experimental conditions. The structural properties and molecular weight characteristics of the twice-RP are comparable to those of the once-RP (Table 1 and S4; entries 15 and 9, Figures S9A, S10, S11A, and S12A), indicating that downcycling is not an issue when the PUFs are subjected to the acidolysis recycling process several times.

## Conclusions

Acidolysis of PUFs with various chemical compositions, including postconsumer PUF waste, was performed with AA at different reaction temperatures, times, and amounts of AA in the VP medium or in bulk. The reaction mixtures were heated with MW, which significantly reduced the reaction time (less than 1 h) compared to PUF acidolysis using conventional heating (several hours). As reaction temperature or time increases, the content of nonhydroxyl end groups of the polyol decreases, but none of the reaction conditions used resulted in a fully hydroxyl-functionalized polyol. With the increasing amount of AA, the content of TDA in the polyol is decreased and the degree of degradation of the urethane groups is improved, but at the expense of a higher degree of esterification of the hydroxyl groups of the polyol by AA, resulting in the RPs being terminated with carboxyl groups to a greater extent. The RPs with different contents of carboxyl and amine end groups were then used for the synthesis of flexible PUFs. With the increasing carboxyl functionality of the RPs in the formulation, the synthesized PUFs show a decreasing average pore size, an increasingly closed-cell morphology, a higher modulus and stress at 40% compression, and a higher compression set, indicating that the carboxyl-terminated polyol strongly affects the quality and performance of the flexible PUFs.

Compared to split-phase glycolysis, which allows complete degradation of urethane groups because glycol is used as the reactant and the medium in a large excess per urethane group and drives the transesterification reaction toward product formation (toluene aminocarbamates, toluene dicarbamates, and free TDA, most of which are soluble in the glycol medium, and an immiscible polyol phase),^25^ acidolysis can be performed with far smaller amounts of the degradation reagent, which is justified by the fact that acidolysis leads to the formation of thermally stable amide bonds, making the acidolysis reaction irreversible. However, with a small excess of degradation reagent per urethane group, acidolysis cannot be carried out to completion because the hydroxyl groups of the polyol may react with the urea groups of the hard segments, resulting in a certain amount of the polyol being terminated with amine groups. Similar to glycolysis, PUF acidolysis performed with a large excess of the degradation reagent results in a high degree of degradation of urethane groups, in the formation of only a small amount of free TDA, and solid di- and monoamidated TDA derivatives that are largely insoluble in the polyol. The main difference, however, is that during acidolysis, esterification as a side reaction inevitably takes place, leading to polyol chains terminated with carboxyl groups. These results indicate that acidolysis of PUF waste cannot produce a recycled polyol that would be a perfect equivalent of the original polyol in terms of polyol functionality. These results suggest that the catalytic system used in the synthesis of flexible PUFs from 100% RPs produced by acidolysis needs to be modified and that knowledge of the polyol functionality, which can be determined by NMR as presented herein, is crucial.

## Supporting Information Available

Experimental conditions and results of characterization of polyols and residues of PUF hard segments by ^1^H NMR, SEC/UV-MALS-RI, MALDI-TOF MS, and FT-IR. Experimental conditions and results of characterization of synthesized flexible PUFs by SEM and DMA (PDF)

## Abbreviations

AA: adipic acid
EO: ethylene oxide
DMA: dynamic mechanical analyzer
EtOAc: ethyl acetate
EtOH: ethanol
FT-IR: Fourier transform infrared spectroscopy
MALDI-TOF MS: matrix-assisted laser desorption/ionization time-of-flight mass spectrometry
MeOH: methanol
MW: microwave
NMR: nuclear magnetic resonance
PO: propylene oxide
PPO: polypropylene oxide
PUF: polyurethane foam
RP: recycled polyol
SEC/UV-MALS-RI: size-exclusion chromatography coupled with ultraviolet, multi-angle light scattering, and refractive index detectors
TDA: toluene diamine
TDI: toluene diisocyanate
VP: virgin polyol
