# Supplementary material for: Insight into Chemical Recycling of Flexible Polyurethane Foams by Acidolysis
Source: ACS Sustain Chem Eng. 2022 Jan 11;10(3):1323–32. doi: 10.1021/acssuschemeng.1c07911 (PMC8790754; doi:10.1021/acssuschemeng.1c07911)
Supplement: Supplementary file 1 — sc1c07911_si_001.pdf [file sc1c07911_si_001.pdf]

Supporting Information

Insight into Chemical Recycling of Flexible Polyurethane Foams by Acidolysis

Maja Grdadolnik,[†] *Ana Drinčić,*[†] *Ana Oreški,*[†] *Ozgun Can Onder,*[†] *Petra Utroša,*[†] *David Pahovnik,*[†] *and Ema Žagar*[†,]*

[†]Department of Polymer Chemistry and Technology, National Institute of Chemistry, Hajdrihova 19, Ljubljana, SI-1000, Slovenia

*Corresponding author: Ema Žagar; Email: ema.zagar@ki.si, Phone: +386 1 4760 203

Number of pages: 20

Number of figures: 13

Number of tables: 4

Table of Contents

List of Tables 2

List of Figures 2

Experimental 4

Materials 4

Characterization methods for polyols 5

Nuclear magnetic resonance (NMR) 5

The non-hydroxyl end group content of the RPs 5

The content of polyol carboxyl (-COOH) and amine (-NH_2) end groups 6

The content of TDA in recycled polyols 6

Size-exclusion chromatography coupled with a multidetection system (SEC/UV-MALS-RI) 7

Matrix-assisted laser desorption / ionization time-of-flight mass spectrometry (MALDI-TOF MS). 7

Fourier-transform infrared spectroscopy (FTIR) 8

Acid value (AV) 8

Hydroxyl number (OH number) 8

The water content 9

Synthesis of flexible PUFs 9

Characterization methods for PUFs 10

Scanning electron microscopy (SEM) 10

Compressive properties 10

Results 11

Results of ^{1}H NMR, SEC/UV-MALS-RI, MALDI-TOF MS, and FTIR characterization of recycled and virgin polyols and residues of PUF hard segments 11

Results of compression testing of synthesized PUFs 20

List of Tables

Table S1. Reaction conditions used for microwave-assisted acidolysis of various PUFs with AA 5

Table S2. Properties of PPO-based VP and its recycled analogs obtained from PUF5611 by acidolysis using 1.1 and 3.0 equivalents of AA per urethane group together with synthesis yields 9

Table S3. The formulations used for synthesis of flexible PUFs. 10

Table S4. Assignation of characteristic signals in ^{1}H NMR spectra. 11

List of Figures

Figure S1. Magnified ^{1}H NMR spectra of residues of hard segments obtained by acidolysis of PUF with 1.1 equivalents of AA per urethane group after 40 min at 210 and 230 °C. The peak assignment refers to the structures in Table S4 13

Figure S2. Magnified ^{1}H NMR spectra of residues of hard segments obtained by acidolysis of PUF with 1.1 equivalents of AA per urethane group at 230 °C after 15 and 40 min. The peak assignment refers to the structures in Table S4 13
Figure S3. Crude reaction mixtures obtained by acidolysis of PUF with AA at 220 °C after 30 min before (A, B, C) and after (D, E, F) centrifugation at 9000 rpm. Acidolysis of homopolyether-based PUF with 1.1 (A, D) and 3.0 (B, E) equivalents of AA per urethane group and acidolysis of copolyether-based PUF with 1.1 equivalents of AA per urethane group (C, F). 14
Figure S4. SEC/UV-MALS-RI chromatograms of a typical upper polyol phase, purified RP and VP of the same type. The solid lines represent the RI detector responses, while the dotted lines represent the UV detector responses recorded at a wavelength of 280 nm. 15
Figure S5. Magnified ^{1}H NMR spectra of purified RPs obtained by PUF acidolysis at 210, 220, 230 °C for 40 min and AA/urethane group ratio of 1.1. ^{1}H NMR spectra were normalized to polyol methyl group at δ 1.04 ppm. Traces of EtOAc used as an extraction solvent and partially amidated 2,4-TDA in RPs are marked with asterisk and h’, respectively. The peak assignment refers to the structures in Table S4 15
Figure S6. Magnified ^{1}H NMR spectra of residues of hard segments obtained after acidolysis of PUF with 1.1, 2.0 and 3.0 equivalents of AA per urethane group at 220 °C, 30 min. The peak assignment refers to the structures in Table S4 16
Figure S7. ^{1}H NMR spectra of polyol upper phases obtained after acidolysis of PUF with 1.1, 2.0 and 3.0 equivalents of AA per urethane group at 220 °C for 30 min. The peak assignment refers to the structures in Table S4 16
Figure S8. A) FTIR spectra and B) SEC/UV-MALS-RI chromatograms of purified RPs obtained after acidolysis of PUF with 1.1 (red), 2.0 (blue) and 3.0 (green) equivalents of AA per urethane group at 220 °C for 30 min together with VP of the same type (black). The solid and dashed lines in B) represent the RI and UV (λ = 280 nm) detector responses, respectively, while the dotted lines represent molecular weight as a function of elution time. 17
Figure S9. A) ^{1}H NMR spectra of purified RPs obtained by acidolysis of PUF5611 in bulk (red) and PUF5611 synthesized from 100 % RP (blue) together with VP of the same type (black). B) ^{1}H NMR spectra of purified RPs obtained by acidolysis of PUF4811 (green) and post-consumer PUF waste (orange) together with VP of the same type (black). 18
Figure S10. MALDI-TOF mass spectra of RPs obtained by acidolysis of PUF5611 in bulk (top) and PUF5611 synthesized from 100 % RP (bottom). The measured monoisotopic signals are denoted in the magnified regions of the mass spectra and are in good agreement with the calculated exact masses (M) ionized with the sodium ion for the proposed structures. 18
Figure S11. A) FTIR spectra of purified RPs obtained by acidolysis of PUF5611 in bulk (red) and PUF5611 synthesized from 100 % RP (blue) together with VP of the same type (black). B) FTIR spectra of purified RPs obtained by acidolysis of PUF4811 (green) and post-consumer PUF waste (orange) together with VP of the same type (black). 19
Figure S12. A) SEC/UV-MALS-RI chromatograms of purified RPs obtained by acidolysis of PUF5611 in bulk (red) and PUF5611 synthesized from 100 % RP (blue) together with VP of the same type (black). B) SEC/UV-MALS-RI chromatograms of purified RPs obtained by acidolysis of PUF4811 (green) and post-consumer PUF waste (orange) together with VP of the same type (black). The solid and dashed lines represent the RI and UV (λ = 280 nm) detector responses, respectively, while the dotted lines represent molecular weight as a function of elution time. 19
Figure S13. Stress-strain curves of PUFs synthesized according to formulations in Table S3 using 100 wt% VP (black), 50 wt% (red) and 100 wt% (green) of RP containing 5.6 mol% of carboxyl and 3.2 mol% of amine functional groups, and 50 wt% (blue) of RP containing 14.0 mol% of carboxyl and 1.6 mol% of amine functional groups. 20

Experimental

Materials

The virgin polyether polyols (VPs) ALCUPOL® F-5611, ALCUPOL® F-4811 and the PUFs prepared from these polyols were supplied by Repsol S.A. ALCUPOL® F-5611 is a trifunctional homopolyether polyol consisting of propylene oxide (PO) repeating units attached to a glycerol core. It has a hydroxyl number of 56 mg KOH g^{-1} and a molecular weight of 3.0 kDa. It contains isomers mixture of (3,5-di-trans-butyl-4-hydroxiphenyl)propionate of C7-9-alkyl (CAS 125643-61-0) as phenolic antioxidant and benzenamine, N-phenyl-, reaction products with 2,4,4-trimethylpentene (CAS 68411-46-1) as amine antioxidant. PUF5611 was synthesized from ALCUPOL® F-5611 (to give 66.1 wt% polyol per PUF), a mixture of 2,4- and 2,6-toluene diisocianate with isomer ratio 80/20 (TDI8020, TDI index of 107), and water as foaming agent to chemically generate CO_2. Kosmos® 29 (tin(II) octoate), TEGOAMIN® 33 and TEGOAMIN® BDE were used as catalysts and silicone TEGOSTAB® BF 2370 was used as a surfactant to control cell size and opening. ALCUPOL® F-4811 is a trifunctional copolyether polyol consisting of PO and ethylene oxide (EO) repeating units attached to a glycerol core, with an EO content of 12 mol% and a molecular weight of 3.5 kDa. It has a hydroxyl number of 48 mg KOH g^{-1} and contains isomers mixture of (3,5-di-trans-butyl-4-hydroxiphenyl)propionate of C7-9-alkyl (CAS 125643-61-0) as phenolic antioxidant and benzenamine, N-phenyl-, reaction products with 2,4,4-trimethylpentene (CAS 68411-46-1) as amine antioxidant. PUF4811 was synthesized from ALCUPOL® F-4811 (to give 65.9 wt% polyol per PUF), a mixture of 2,4- and 2,6-toluene diisocianate with an isomer ratio of 80/20 (TDI8020, TDI index of 107), and water as a foaming agent to chemically generate CO_2. Kosmos® 29, TEGOAMIN® 33 and TEGOAMIN® BDE were used as catalysts and silicone TEGOSTAB® BF 2370 was used as surfactant in PUF4811 synthesis.

Adipic acid (AA), imidazole and pyridine were purchased from Sigma Aldrich (Darmstadt, Germany); phenolphthalein and phthalic anhydride were purchased from Merck (Darmstadt, Germany); potassium hydrogen phthalate (KHP) was purchased from Acros Organics (USA); hydrochloric acid (HCl) and deuterated dimethyl sulfoxide (DMSO-d_6) were purchased from Euriso Top (Saarbrücken, Germany); methanol (MeOH) from J.T. Baker (USA); trifluoroacetic acid (TFA, 99 %) from Aldrich (Darmstadt, Germany); ethanol (EtOH) from Carlo Erba (Italy); chloroform ($CHCl_3$), ethyl acetate (EtOAc) and sodium hydroxide (NaOH) from Honeywhell (Charlotte, North Carolina, USA); Aquastar water standard 0.01 %

and Aquastar CombiCoulomat reagent were purchased from Supelco (Darmstadt, Germany). TDI Ongronate TDI (80/20), Kosmos® 29 (tin(II) octoate catalyst), B11 (tertiary amine catalyst) and silicone 2370 were supplied by Plama-pur. All chemicals were used as received.

Table S1. Reaction conditions used for microwave-assisted acidolysis of various PUFs with AA.

**Entry**	**PUF type**	***m*(PUF) (g)**	***m*(VP) (g)**	**AA/urethane group (mol/mol)**	***T* (°C)**	***t* (min)**
1	PUF5611	6	3	1.1	210	15
2	PUF5611	6	3	1.1	210	30
3	PUF5611	6	3	1.1	210	40
4	PUF5611	6	3	1.1	220	15
5	PUF5611	6	3	1.1	220	30
6	PUF5611	6	3	1.1	220	40
7	PUF5611	6	3	1.1	230	15
8	PUF5611	6	3	1.1	230	30
9	PUF5611	6	3	1.1	230	40
10	PUF5611	6	3	2.0	220	30
11	PUF5611	6	3	3.0	220	30
12	PUF5611-bulk	4	/	1.1	230	30
13	PUF4811	6	3	1.1	220	30
14	Post-Consumer PUF	6	3	1.1	220	30
15	Recycled PUF5611	6	3	1.1	230	40

Characterization methods for polyols

Nuclear magnetic resonance (NMR)

^{1}H NMR spectra were recorded using a Bruker AVANCE NEO 600 MHz instrument (Bruker Corporation, USA) at room temperature in DMSO-d_6 with or without the addition of TFA. Chemical shifts are given in ppm relative to a DMSO-d_6 residual peak.

The non-hydroxyl end group content of the RPs

The non-hydroxyl end group content of the RPs was determined from ^{1}H NMR spectra recorded in DMSO-d_6 with added TFA to shift the signal of the amine groups, which overlaps

with that of the polyol methyne groups, to a lower magnetic field. The non-hydroxyl end group content of the RPs was determined according to equation S1 from the integral of the overlapping signals of the polyol methyne protons adjacent to the urethane and ester groups at δ 4.88 ppm (c' and r), and the integral of the methyl signal (–C$\underline{H}_3$; a) of the polyol at δ 1.04 ppm, assuming 50 PO repeating units in the polyol and polyol functionality of 3.

$$\textbf{\textit{Non-hydroxyl end group content}}\ (\%) = \frac{I(>CH-)_{c'+r} \times 50}{I(-CH_3)_{PO}} \times 100 \qquad (S1)$$

The content of polyol carboxyl (-COOH) and amine (-NH$_2$) end groups

The content of carboxyl (-COOH) and amine (-NH$_2$) end groups was determined from ^{1}H NMR spectra of the purified RPs recorded in DMSO-d_6. The content of carboxyl groups was determined according to equation S2 based on the intensity of the proton signals at δ 2.20 and 2.26 ppm (denoted as p and p′) corresponding to the methylene groups of the AA residue adjacent to the carboxyl and ester groups, respectively, and the intensity of the methyl signal (a) of the polyol at δ 1.04 ppm. The content of amine functional groups of the purified polyols was determined according to equation S3 from the intensity of the methyl signals of the aromatic amine end groups attached to the polyol via the urethane groups (δ 2.00, 1.96 and 1.87 ppm for both isomers; denoted as d) and the intensity of the methyl signal (a) of the polyol at δ 1.04 ppm, assuming 50 PO repeating units in the polyol and polyol functionality of 3.

$$\textbf{\textit{-COOH content}}\ (\%) = \frac{I(-CH_2-)_{p+p'} \times 50}{I(-CH_3)_{PO} \times 4} \times 100 \qquad (S2)$$

$$\textbf{\textit{-NH}}_2\ \textbf{\textit{content}}\ (\%) = \frac{I(-CH_3)_{d} \times 50}{I(-CH_3)_{PO} \times 3} \times 100 \qquad (S3)$$

The content of TDA in recycled polyols

The content of TDA in unpurified RPs (upper polyol phases) was determined from ^{1}H NMR spectra recorded in DMSO-d_6 according to equation S4 from the signal intensities of the methyl groups of the TDA isomers (δ 1.79 for 2,6-TDA and 1.88 ppm for 2,4-TDA; signals e & h) and the methyl group (a) of PO repeating unit of the polyol at δ 1.04 ppm.

$$\textbf{\textit{TDA content}}\ \textbf{(wt\%)} = \frac{I(-CH_3)_{\mathrm{TDA}} \times \mathrm{M(TDA)}}{\left(I(-CH_3)_{\mathrm{PO}} \times \mathrm{M(PO)} + I(-CH_3)_{\mathrm{TDA}} \times \mathrm{M(TDA)}\right)} \times 100 \qquad \text{(S4)}$$

Size-exclusion chromatography coupled with a multidetection system (SEC/UV-MALS-RI)

The molecular weight characteristics (M_w, M_n, dispersity: M_w/M_n) and purity of the RPs were determined using the chromatographic system SEC/UV-MALS-RI, which consists of an HPLC pump, a degasser and an autosampler (Agilent Technologies, USA). The HPLC system is coupled with detectors connected in series: a UV detector operating at a wavelength of 280 nm (Agilent Technologies, USA), a DAWN multi-angle light-scattering photometer (Wyatt Technology Corp., USA) and an Optilab interferometric refractometer (RI) (Wyatt Technology Corp., USA). Separations were performed at room temperature using a TSKgel Alpha-2500 SEC column (7.8 mm ID × 30.0 cm L, pore size 7 µm, and exclusion limit 10 kDa) with a precolumn (Tosoh Bioscience GmbH, Germany). Methanol was used as the solvent and mobile phase with a flow rate of 0.7 mL min^{-1}. Toluene was used to calibrate the 90 ° LS detector, while the other detectors were normalized with the polyethylene oxide standard with a weight average molecular weight of 4 kDa and dispersity of 1.02. The masses of the samples injected onto the column were typically 1.0×10^{-3} g, while the concentrations of the solution were typically 1.0×10^{-2} g mL^{-1}. The specific refractive index increment (dn/dc) required for the calculation of molecular weights was determined assuming 100 % mass recovery of the ALCUPOL® F-5611 or ALCUPOL® F-4811 polyols from the column. Astra 8 software was used for data acquisition and analysis (Wyatt Technology Corp., USA).

Matrix-assisted laser desorption / ionization time-of-flight mass spectrometry (MALDI-TOF MS)

MALDI-TOF MS measurements were performed using a Bruker UltrafleXtreme MALDI-TOF mass spectrometer (Bruker Daltonics, Germany). Polyol samples were dissolved in THF (10 mg mL^{-1}) and mixed with a solution of the matrix, 2,5-dihydroxybenzoic acid in THF (30 mg mL^{-1}) and sodium trifluoroacetate in THF (10 mg mL^{-1}), in a volume ratio of 1:10:3. A 0.4 µL of the prepared solution was spotted onto the target plate (dried-droplet method). The mass spectra of the samples were recorded in reflective positive ion mode. Calibration was performed externally using a mixture of poly(methyl methacrylate) standards dissolved in THF (MALDI validation set PMMA, Fluka Analytical) that covered the measured molecular weight range. Sample preparation for the standard mixture was the same as for the samples. The standard mixture was spotted to the nearest neighbor positions.

Fourier-transform infrared spectroscopy (FTIR)

FTIR transmission spectra were recorded using a Spectrum One FTIR spectrometer (Perkin-Elmer, Waltham, MA, USA) in ATR mode in a spectral range of 400–4000 cm^{-1} with a spectral resolution of 4 cm^{-1}.

Acid value (AV)

AV was determined according to the adapted standard ASTM D4662-08 (Reapproved 2011), using only ethanol as solvent instead of a mixture of toluene and ethanol. Approximately 1 g of polyol was dissolved in 5 mL of ethanol. A solution of phenolphthalein (50 µL) in ethanol was used as a color indicator. Titrations were performed using 0.01 N NaOH solution in triplicate to determine the average value. The end point of the titration was determined visually by a change in color of the solution to pink. The AV was calculated using equation S5.

$$\boldsymbol{AV} = \frac{(A-B) \times 56.1 \times N}{w} \tag{S5}$$

where *A* is the volume of NaOH solution (mL) required to titrate the sample; *B* is the volume of NaOH solution (mL) required to titrate the blank; *N* is the normality of the NaOH solution, and *w* is the weight of the sample (g).

Hydroxyl number (OH number)

The OH number was determined according to ASTM D4274-05 standard, where the esterification process of the polyol with phthalic anhydride was catalyzed by imidazole. Phenolphthalein dissolved in pyridine (50 µL) was used as the color indicator. An esterification reagent (phthalic anhydride) was used as a blank. Titrations with an aqueous 0.5 N NaOH solution were performed in triplicate to determine the average value. The end point of the titration was determined visually by a change in color of the solution to pink. The OH number was calculated according to equation S6,

$$\boldsymbol{OH}_{\mathbf{number}} = \frac{(B-A) \times 56.1 \times N}{w} \tag{S6}$$

where *A* is the volume of NaOH solution (mL) required to titrate the sample; *B* is the volume of NaOH solution (mL) required to titrate the blank; *N* is the normality of the NaOH solution and *w* is the weight of the sample (g).

The OH number was corrected considering AV and calculated according to equation S7.

$$\boldsymbol{Corrected\ OH}_{\mathbf{number}} = \frac{(B-A) \times 56.1 \times N}{w} + AV \tag{S7}$$

The water content

The water content in the polyols was determined by the adapted standard method ASTM D4672 12, in which chloroform was used as an additional solvent to improve the solubility of the polyols. Karl Fischer titration was performed using a C10S Compact KF coulometer (Mettler Toledo, Columbus, USA).

Synthesis of flexible PUFs

Table S2. Properties of PPO-based VP and its recycled analogs obtained from PUF5611 by acidolysis using 1.1 and 3.0 equivalents of AA per urethane group together with synthesis yields.

**Sample**	**OH number (mg KOH g^{-1})**	**Acid value (mg KOH g^{-1})**	**Corrected OH number (mg KOH g^{-1})**	**Water content (ppm)**	**-COOH content (mol %)**	**-NH_2 content (mol %)**	**M_w (g/mol)**	**Đ**	***Yield**** **(%)**	***Yield***** **(%)**
VP	55.97	0.05	56.02	296	/	/	2970	1.02	/	/
RP-1.1Eq AA	55.76	2.41	58.17	119	5.6	3.2	3160	1.02	91	60.2
RP-3.0Eq AA	48.40	7.11	55.51	1162	14.0	1.6	3270	1.02	90	59.5

* Determined based on theoretical content of the polyol in the PUF (66.1 wt%) after polyol purification. ** Yield (w/w) of the obtained RPs related to the PUF.

Table S3. The formulations used for synthesis of flexible PUFs. The quantities are given in "part per polyol" (ppp; w/w).

PUF Denotation	VP	RP	dH_2O	B11	Kosmos® 29	Silicone BF 2470	TDI 80/20	TDI Index
VP (ALCUPOL® F-5611)	100	0	4.5	0.15	0.18	1.2	56.16	107
AC1.1-RP50	50	50	4.5	0.15	0.18	1.2	56.16	107
AC1.1-RP100	0	100	4.5	0.15	0.18	1.2	56.16	107
AC3.0-RP50	50	50	4.5	0.15	0.18	1.2	56.16	107

Characterization methods for PUFs

Scanning electron microscopy (SEM)

The morphology of the PU foams was studied by scanning electron microscopy (SEM) on a high-resolution SEM Zeiss Ultra plus instrument (Carl Zeiss, Germany). The foams were cut with a razor blade perpendicular to the foam rise direction. For charge dissipation during SEM analysis, the obtained cross-sections were coated with a 10 nm thick gold layer using a Gatan PECS 682 (Gatan, USA). Pore size analysis was performed with ImageJ software using at least 100 pores from three different cross-sections.

Compressive properties

The compressive properties of the PU foams were determined using a DMA Q800 dynamic mechanical analyzer (TA Instruments) and 40 mm diameter compression discs. The specimens were cut into a rectangular cuboid shape (10 mm height, 25 mm width, 25 mm length) and compressed at 50 % min^{-1} up to 70 % of the original specimen height and then decompressed at the same rate to the original specimen height. The procedure was repeated three times and at the fourth compression cycle, the stress-strain curve was recorded from which the Young's modulus was determined from the initial linear range and the stress at 40 % strain. The average values of three specimens with standard errors are given for each sample.

To determine the compression set, the specimens with rectangular cuboid shape (10 mm height, 25 mm width, 25 mm length) were compressed to 50 % of the initial height (d_0) and heated to 70 °C. After 22 h, the foams were allowed to recover for 30 min at ambient

conditions. The height (d_r) was measured again and the compression set, expressed as a percentage, is calculated according to equation S8:

$$\textit{\textbf{Compression set}}\ \mathbf{(\%)} = \frac{d_0 - d_r}{d_0} \times 100 \qquad \text{(S8)}$$

where d_0 and d_r are the heights of the original specimen and the specimen after compression testing, respectively.

Results

Results of ^{1}H NMR, SEC/UV-MALS-RI, MALDI-TOF MS, and FTIR characterization of recycled and virgin polyols and residues of PUF hard segments

Table S4. Assignation of characteristic signals in ^{1}H NMR spectra.

Compound	Molecular Structure	Characteristic Chemical Shift(s), ppm
PPO-based polyol	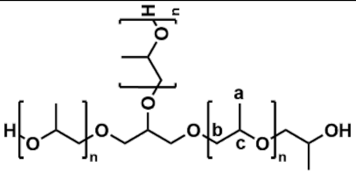	**a**; 1.04 ppm<br>**b**, **c**; 3.15–3.70 ppm<br>-O**H**; 4.40 ppm
Amine functionalized PPO-polyol	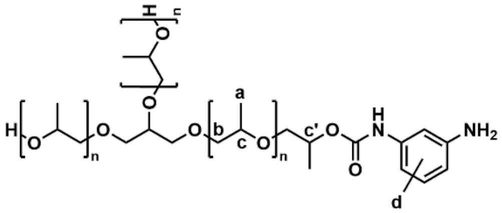	**d**; 1.87, 1.96, 2.00 ppm<br>**c'**; 4.88 ppm (DMSO-d_6 + TFA)<br>-N**H**COO-; 9.18; 8,58; 8,47 ppm<br>-N**H**$_2$; 4.70–4.90 ppm
Adipic acid functionalized PPO-polyol	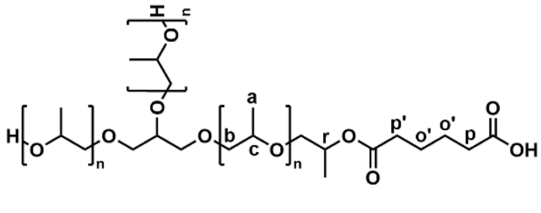	**o'**; 1.51 ppm<br>**p;** 2.20 ppm<br>**p';** 2.26 ppm<br>**r;** 4.88 ppm (DMSO-d_6 + TFA)<br>-COO**H**; 12.00 ppm
2,6-toluene diamine (2,6-TDA)	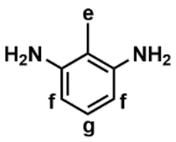	**e**; 1.79 ppm<br>**f**; 5.92 ppm<br>**g**: 6.54 ppm<br>-N**H**$_2$; 4.00–4.50 ppm
2,4-toluene diamine (2,4-TDA)	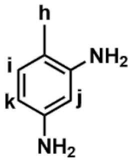	**h**; 1.88 ppm<br>**i**; 6.54 ppm<br>**j**; 5.88 ppm<br>**k**; 5.75 ppm<br>-N**H**$_2$; 4.00–4.50 ppm

Partially amidated 2,6-TDA	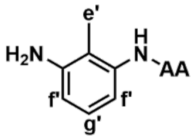	**e’**; 1.86 ppm<br>**f’, g’**; 6.00–7.00 ppm<br>-N**H**CO-; 8.95–9.88 ppm<br>-N**H**$_2$; 4.70–4.90 ppm
Partially amidated 2,4-TDA	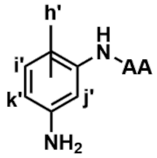	**h’**; 1.97 ppm<br>**i’, j’, k’**; 6.00–7.00 ppm<br>-N**H**CO-; 8.95–9.88 ppm<br>-N**H**$_2$; 4.70–4.90 ppm
Fully amidated 2,6-TDA	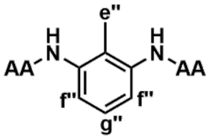	**e’’**; 2.02 ppm<br>**f’’, g’’**; 7.00–7.60 ppm<br>-N**H**CO-; 8.95–9.88 ppm
Fully amidated 2,4-TDA	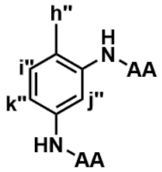	**h’’**; 2.11 ppm<br>**i’’, j’’, k’’**; 7.00–7.60 ppm<br>-N**H**CO-; 8.95–9.88 ppm
Amine terminated (oligo)urea	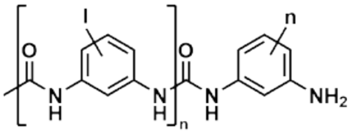	**n;** 1.94, 2.03–2.09 ppm<br>**l**; 2.17 and 2.20 ppm<br>N**H**$_2$; 4.70–4.90 ppm
Adipic acid (AA)	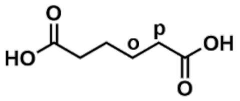	**o**; 1.50 ppm<br>**p**; 2.20 ppm<br>-COO**H**; 12.00 ppm
Terminal amidated aromatic isomers of the oligourea segments	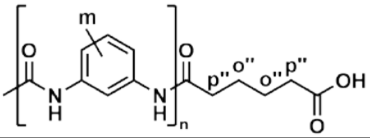	**m**; 2.08 ppm<br>**o’’**; 1.53–1.72 ppm<br>**p’’**; 2.22–2.39 ppm<br>-COO**H**; 12.00 ppm

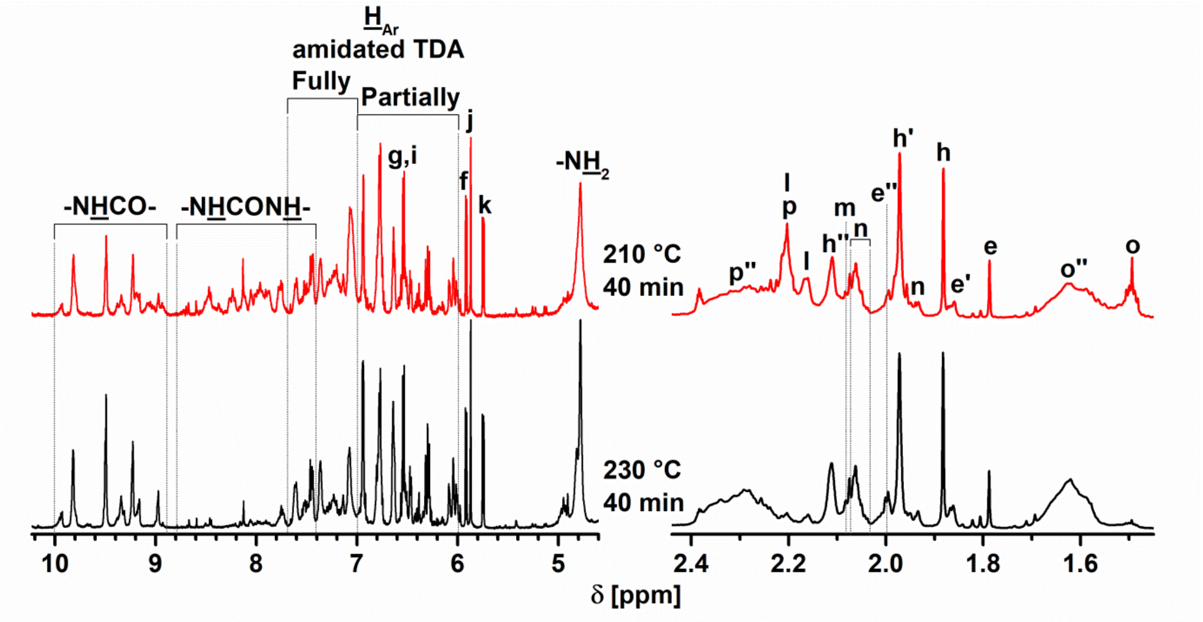

Figure S1. Magnified ^{1}H NMR spectra of residues of hard segments obtained by acidolysis of PUF with 1.1 equivalents of AA per urethane group after 40 min at 210 and 230 °C. The peak assignment refers to the structures in Table S4.

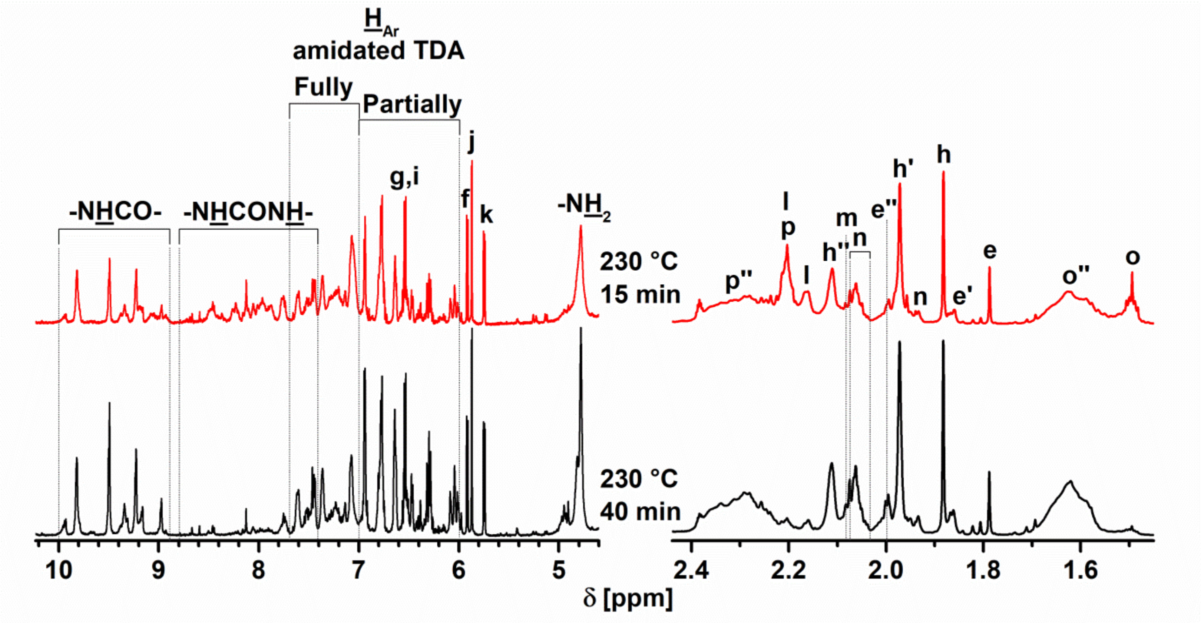

Figure S2. Magnified ^{1}H NMR spectra of residues of hard segments obtained by acidolysis of PUF with 1.1 equivalents of AA per urethane group at 230 °C after 15 and 40 min. The peak assignment refers to the structures in Table S4.

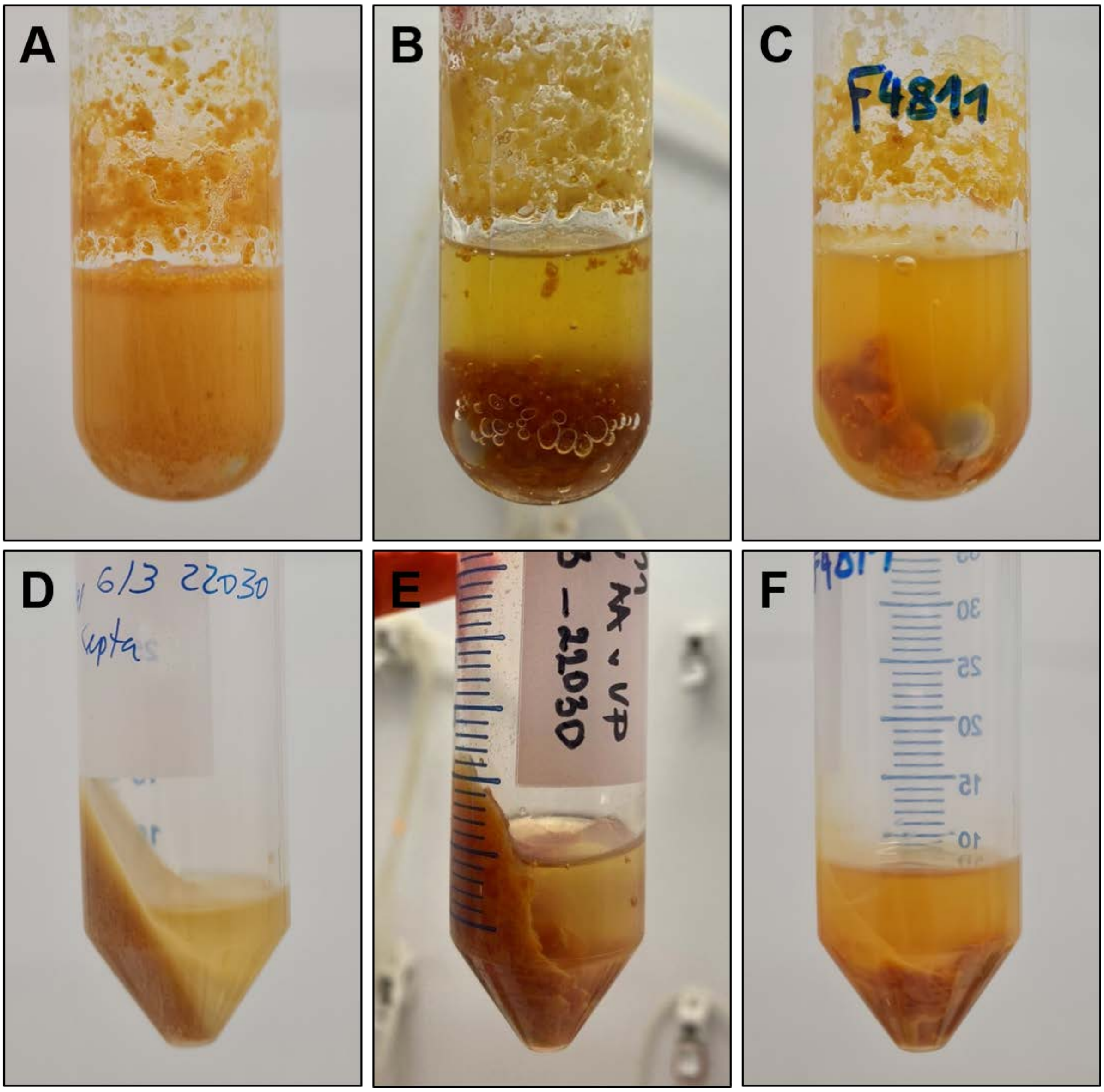

Figure S3. Crude reaction mixtures obtained by acidolysis of PUF with AA at 220 °C after 30 min before (A, B, C) and after (D, E, F) centrifugation at 9000 rpm. Acidolysis of homopolyether-based PUF with 1.1 (A, D) and 3.0 (B, E) equivalents of AA per urethane group and acidolysis of copolyether-based PUF with 1.1 equivalents of AA per urethane group (C, F).

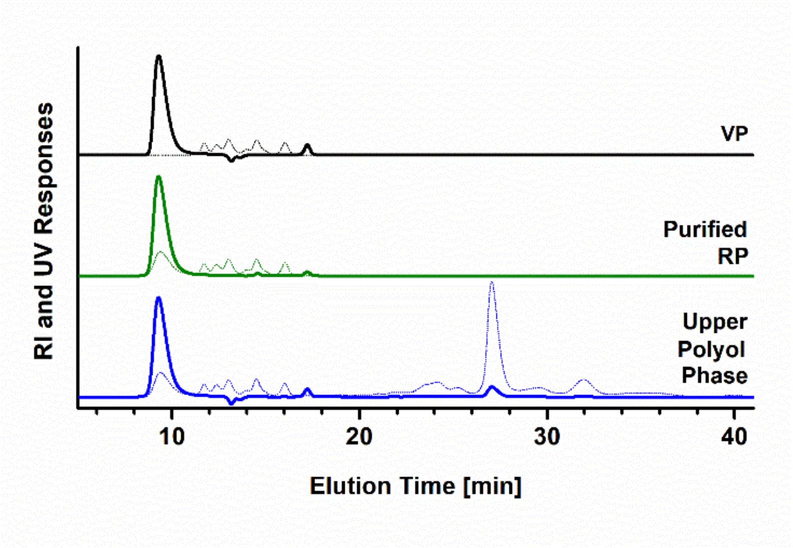

Figure S4. SEC/UV-MALS-RI chromatograms of a typical upper polyol phase, purified RP and VP of the same type. The solid lines represent the RI detector responses, while the dotted lines represent the UV detector responses recorded at a wavelength of 280 nm.

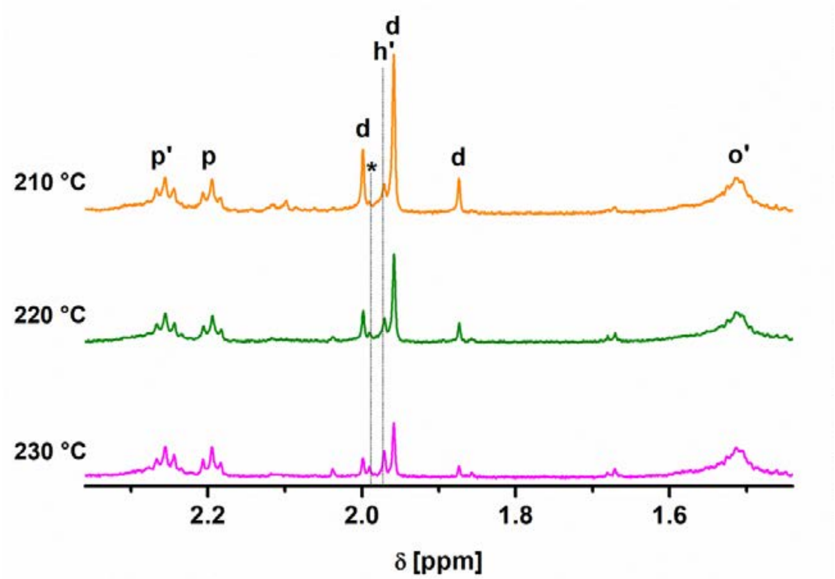

Figure S5. Magnified ^{1}H NMR spectra of purified RPs obtained by PUF acidolysis at 210, 220, 230 °C for 40 min and AA/urethane group ratio of 1.1. ^{1}H NMR spectra were normalized to polyol methyl group at δ 1.04 ppm. Traces of EtOAc used as an extraction solvent and partially amidated 2,4-TDA in RPs are marked with asterisk and h', respectively. The peak assignment refers to the structures in Table S4.

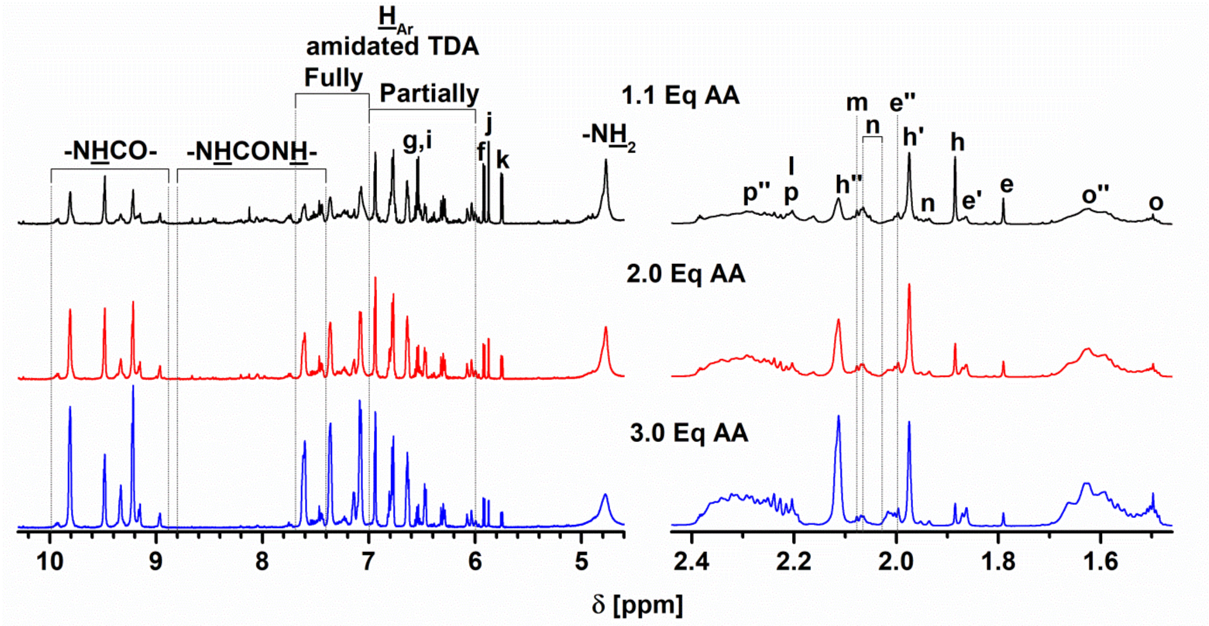

Figure S6. Magnified ^{1}H NMR spectra of residues of hard segments obtained after acidolysis of PUF with 1.1, 2.0 and 3.0 equivalents of AA per urethane group at 220 °C, 30 min. The peak assignment refers to the structures in Table S4.

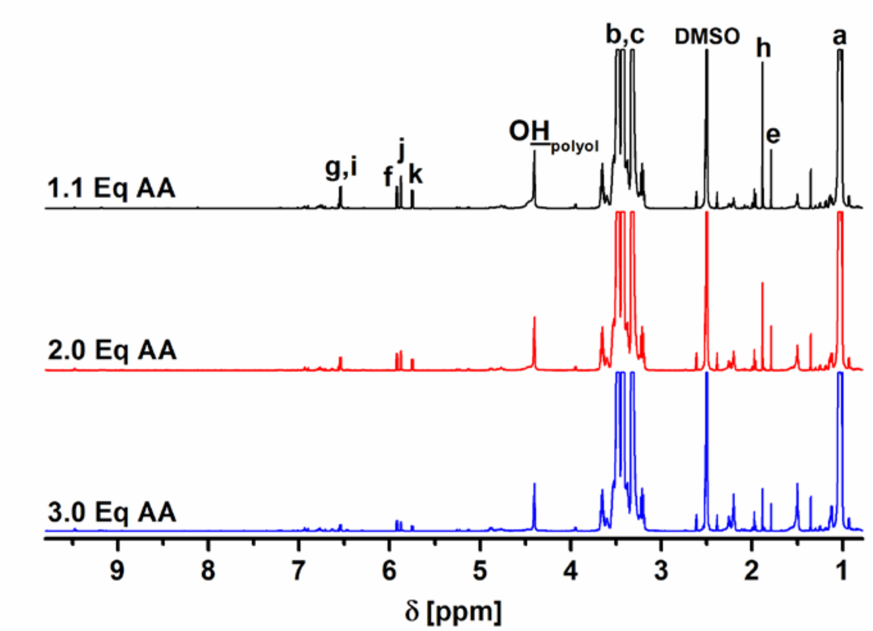

Figure S7. ^{1}H NMR spectra of polyol upper phases obtained after acidolysis of PUF with 1.1, 2.0 and 3.0 equivalents of AA per urethane group at 220 °C for 30 min. The peak assignment refers to the structures in Table S4.

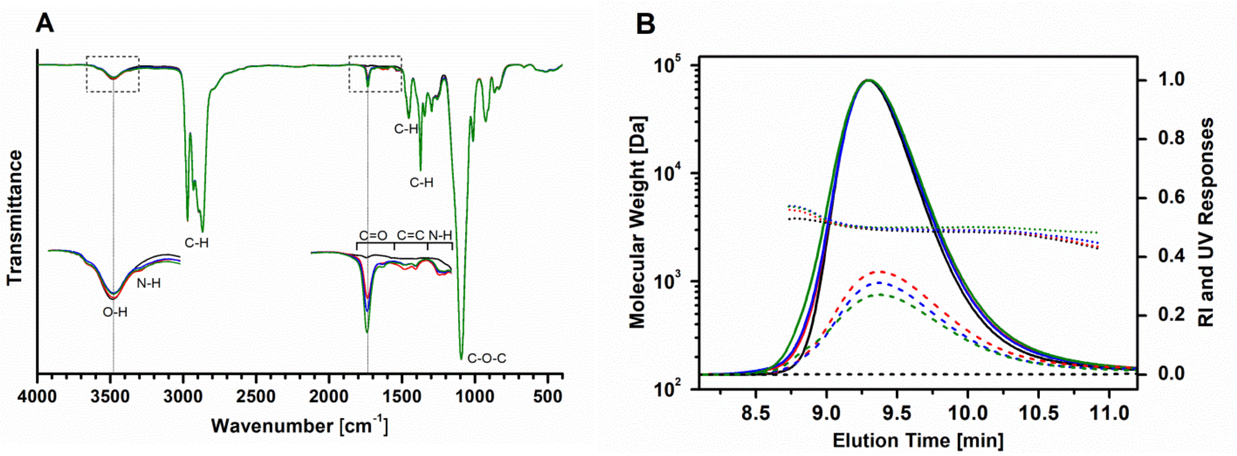

Figure S8. A) FTIR spectra and B) SEC/UV-MALS-RI chromatograms of purified RPs obtained after acidolysis of PUF with 1.1 (red), 2.0 (blue) and 3.0 (green) equivalents of AA per urethane group at 220 °C for 30 min together with VP of the same type (black). The solid and dashed lines in B) represent the RI and UV (λ = 280 nm) detector responses, respectively, while the dotted lines represent molecular weight as a function of elution time.

The broad band centered at 3474 cm^{-1} in FTIR spectra represents the O–H stretching vibration of polyol hydroxyl and carboxyl end groups. The shoulder at 3367 cm^{-1} is due to N–H stretching vibration of polyol amine end groups. The bands between 3060 and 2750 cm^{-1} correspond to C–H stretching vibrations and the bands centered at 1454 cm^{-1} and 1373 cm^{-1} to C–H bending vibrations of the polyol methyl, methylene and methyne groups. The intense band at 1094 cm^{-1} is due to C–O–C stretching vibration of polyol ether groups. The band centered at 1733 cm^{-1} is due to C=O stretching vibration of urethane, ester and carboxyl groups in the polyol structure. Its intensity increases with increasing amount of AA used to degrade the PUF. The bands in the 1650–1580 cm^{-1} and 1560–1505 cm^{-1} regions correspond to C=C vibration of aromatic rings and N–H bending vibration, respectively.

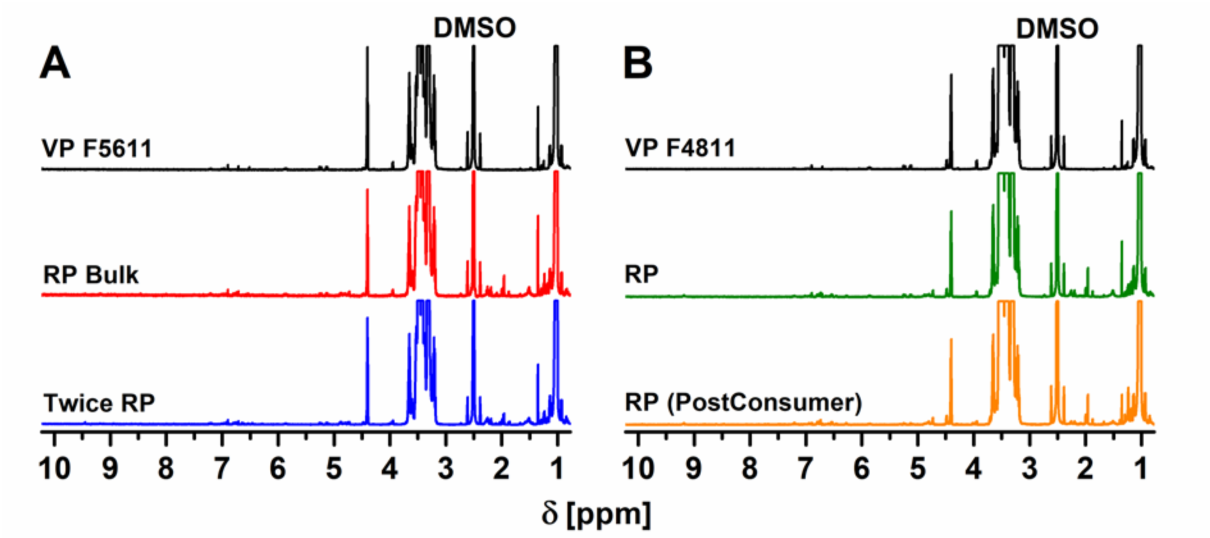

Figure S9. A) ^{1}H NMR spectra of purified RPs obtained by acidolysis of PUF5611 in bulk (red) and PUF5611 synthesized from 100 % RP (blue) together with VP of the same type (black). B) ^{1}H NMR spectra of purified RPs obtained by acidolysis of PUF4811 (green) and post-consumer PUF waste (orange) together with VP of the same type (black).

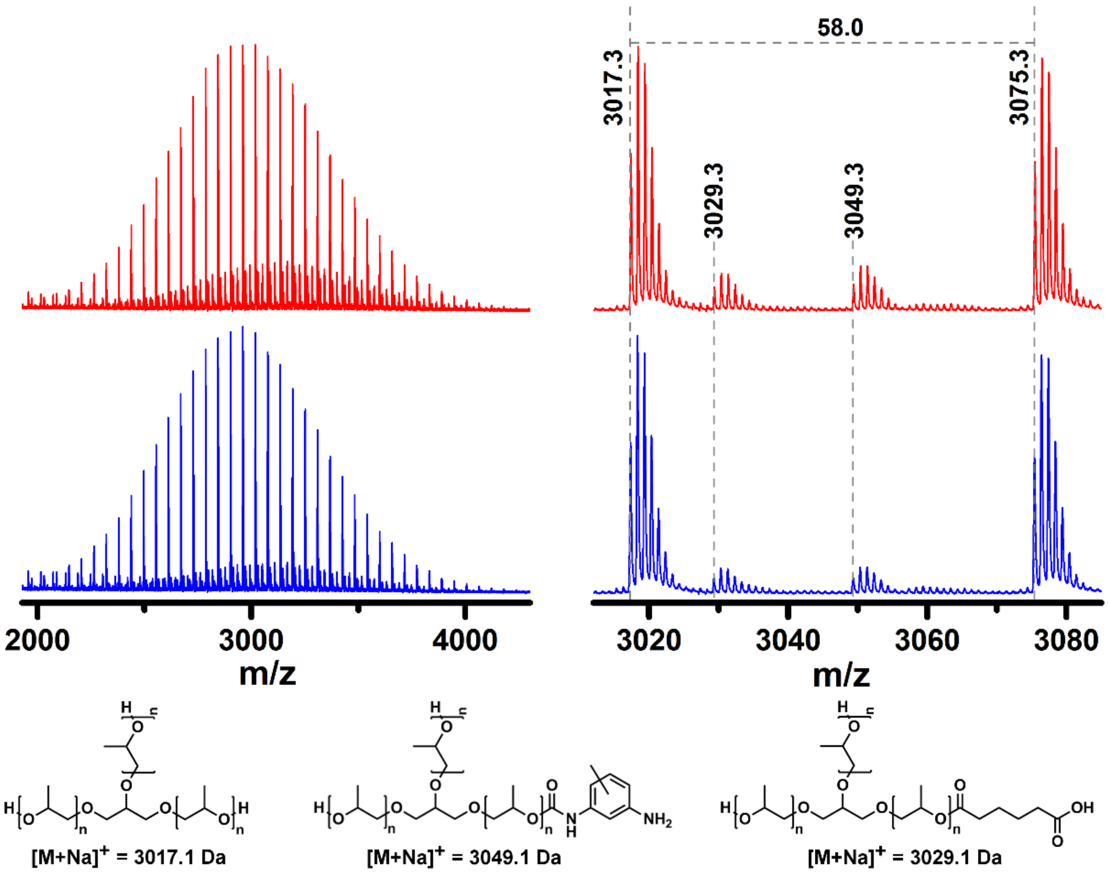

Figure S10. MALDI-TOF mass spectra of RPs obtained by acidolysis of PUF5611 in bulk (top) and PUF5611 synthesized from 100 % RP (bottom). The measured monoisotopic

signals are denoted in the magnified regions of the mass spectra and are in good agreement with the calculated exact masses (M) ionized with the sodium ion for the proposed structures.

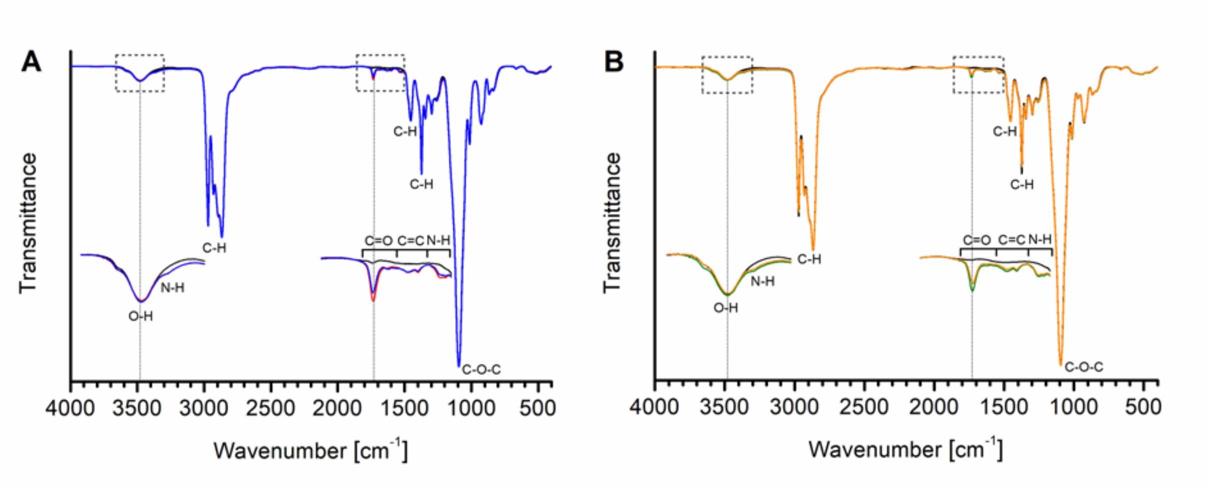

Figure S11. A) FTIR spectra of purified RPs obtained by acidolysis of PUF5611 in bulk (red) and PUF5611 synthesized from 100 % RP (blue) together with VP of the same type (black). B) FTIR spectra of purified RPs obtained by acidolysis of PUF4811 (green) and post-consumer PUF waste (orange) together with VP of the same type (black).

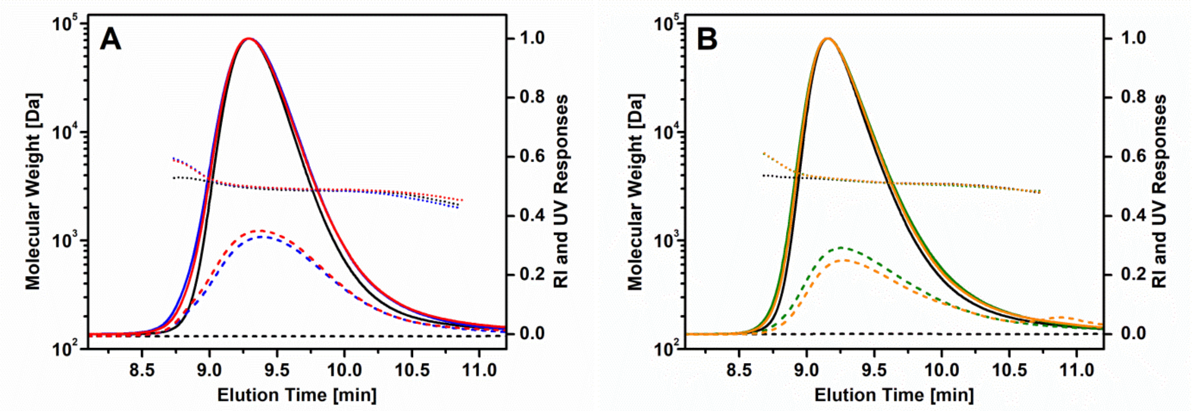

Figure S12. A) SEC/UV-MALS-RI chromatograms of purified RPs obtained by acidolysis of PUF5611 in bulk (red) and PUF5611 synthesized from 100 % RP (blue) together with VP of the same type (black). B) SEC/UV-MALS-RI chromatograms of purified RPs obtained by acidolysis of PUF4811 (green) and post-consumer PUF waste (orange) together with VP of the same type (black). The solid and dashed lines represent the RI and UV (λ = 280 nm) detector responses, respectively, while the dotted lines represent molecular weight as a function of elution time.

Results of compression testing of synthesized PUFs

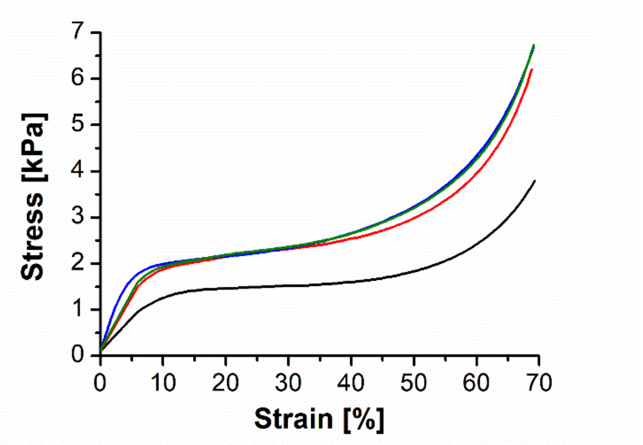

Figure S13. Stress-strain curves of PUFs synthesized according to formulations in Table S3 using 100 wt% VP (black), 50 wt% (red) and 100 wt% (green) of RP containing 5.6 mol% of carboxyl and 3.2 mol% of amine functional groups, and 50 wt% (blue) of RP containing 14.0 mol% of carboxyl and 1.6 mol% of amine functional groups.
